# Breast Cancer Detection Using Infrared Thermography: A Survey of Texture Analysis and Machine Learning Approaches

**DOI:** 10.3390/bioengineering12060639

**Published:** 2025-06-11

**Authors:** Larry Ryan, Sos Agaian

**Affiliations:** Department of Computer Science, Graduate Center, CUNY, City University of New York, New York, NY 10016, USA; sos.agaian@csi.cuny.edu

**Keywords:** thermography, breast cancer, texture, image processing, medical image analysis

## Abstract

Breast cancer remains a leading cause of cancer-related deaths among women worldwide, highlighting the urgent need for early detection. While mammography is the gold standard, it faces cost and accessibility barriers in resource-limited areas. Infrared thermography is a promising cost-effective, non-invasive, painless, and radiation-free alternative that detects tumors by measuring their thermal signatures through thermal infrared radiation. However, challenges persist, including limited clinical validation, lack of Food and Drug Administration (FDA) approval as a primary screening tool, physiological variations among individuals, differing interpretation standards, and a shortage of specialized radiologists. This survey uniquely focuses on integrating texture analysis and machine learning within infrared thermography for breast cancer detection, addressing the existing literature gaps, and noting that this approach achieves high-ranking results. It comprehensively reviews the entire processing pipeline, from image preprocessing and feature extraction to classification and performance assessment. The survey critically analyzes the current limitations, including over-reliance on limited datasets like DMR-IR. By exploring recent advancements, this work aims to reduce radiologists’ workload, enhance diagnostic accuracy, and identify key future research directions in this evolving field.

## 1. Introduction

Breast cancer is the most commonly diagnosed cancer among women and has the highest cancer-related mortality rate [1]. Although the mortality rate in high-income countries dropped by 40% between the 1980s and 2020 due to improved access to treatment centers equipped for cancer diagnosis and effective treatment [2], globally, 2.3 million new cases and 670,000 women died from breast cancer in 2022 [3].

Early detection is essential to decrease mortality rates and improve survival rates. Self-breast exam (SBE) can be performed by the patient but has a low ability to detect breast cancer (sensitivity). Mammography is the primary modality recommended by the FDA and is widely used in wealthier countries [4]. However, it is less effective for dense breasts, has a higher false positive rate [5], causes discomfort, and utilizes ionizing radiation which slightly increases cancer risk with repeated exposures [6,7]. Other modalities that are employed as an adjunct to mammography are Magnetic Resonance Imaging (MRI) and ultrasound. MRI has greater sensitivity than mammography, but is expensive [8]. Ultrasound helps characterize benign cysts and determine whether a solid mass is benign or malignant [9]. Table 1 summarizes these modalities and compares them with thermography. These modalities are also described and compared in Prasad and Houserkova [10].

However, in developing countries, access to mammography is limited due to costs, cultural barriers, a shortage of trained personnel, and inadequate medical infrastructure [13,14]. The Breast Health Global initiative of the World Health Organization (WHO) has recommended that developing countries adopt Clinical Breast Exam (CBE) [15]; however, its efficacy in detecting breast cancer in an early stage is very low (40–69% sensitivity) when compared to mammography 77–95% [16,17,18]. Therefore, there is a gap that requires the adoption of an alternative modality [19].

**Figure 1:**
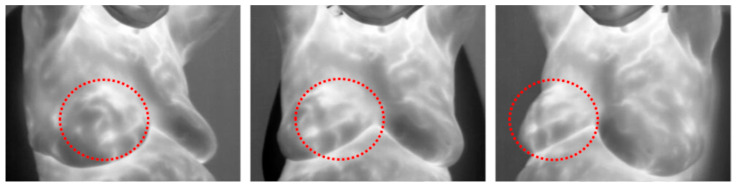
Right oblique, frontal, and left oblique images of patient IIR0035 from the Mendeley dataset [21]. The red dotted circle shows the thermal pattern of a cancerous lesion in the patient’s right breast. The patient’s left breast is normal. Note that there is a brighter more elaborate pattern in the patient’s right breast.

Thermography is a non-invasive, non-X-ray modality significantly more cost-effective than mammography. It replaces the expensive X-ray machine with a more affordable infrared camera. Thermography detects infrared radiation emitted by the skin’s surface, which includes reflected heat and heat generated by subcutaneous tissues. Due to the higher metabolic activity of cancer lesions, the subcutaneous tissue and skin temperature increase. This heightened metabolic activity leads to angiogenesis, the formation of new blood vessels, and vasodilation, the dilation of blood vessels [6]. The combined effects of increased metabolism and greater blood flow raise the temperature of the surrounding tissue, which is detectable by an infrared camera (See Figure 1 below). Because thermography can detect these changes in the early stages of cancer, it has the potential to detect cancer earlier than mammography [6]. It is suitable for females of any age, from pre-adolescence to postmenopausal, including those with dense or fibrocystic cystic breast tissues, as well as pregnant or breastfeeding women, since it does not involve any radiation exposure like mammography. However, distinguishing the underlying cause of a temperature increase in the breast can be challenging, as it may result from cancer or an alternative cause such as bacterial or viral [20]. This difficulty may explain thermography’s higher specificity.

Thermography could be a more suitable alternative for developing countries due to its lower cost than mammography and greater effectiveness relative to self-breast examinations (SBE) and clinical breast examinations (CBE). However, radiologists struggle with interpreting thermography images and could use help analyzing them. Furthermore, one study reported that the average radiologist must interpret one image every 3–4 s in an 8-h workday to meet workload demands [22]. A computer-aided diagnostic (CAD) system that incorporates texture analysis and machine learning can assist them in locating and segmenting the abnormal region, classifying the type of abnormality, and assigning a confidence score to assess cancer risk.

When detecting abnormalities in thermographic images of breasts, the temperature pattern appears as an area of increased temperature distribution (see dashed red circle in Figure 1). Texture analysis involves recognizing these patterns and extracting features that enable accurate classification by a machine learning model. These arrangements and structures of similar elements are called texels or textons [23]. Therefore, texture analysis would appear to be a valuable approach for extracting discriminant features of breast cancer lesions.

The aim of this paper is to provide a comprehensive literature review of the use of texture analysis and machine learning for the classification of thermographic images. Ghalati et al. [24] review the application of texture analysis in biomedical imaging across multiple diseases and modalities, but excludes thermography. There are several reviews [25,26,27,28] covering the development of computer-aided diagnostic (CAD) systems for the detection of breast cancer via thermography, which predominantly focus on neural-network-based feature extraction and classification. However, no existing review specifically focuses on the extraction of texture features from thermograms for the detection of cancer in breasts. Although neural network-enhanced CAD systems perform well, this review shows that texture features-based CAD systems perform equally as well, and can enhance the performance of neural networks [29]. Furthermore, texture-based CAD systems can be trained on smaller datasets, are computationally more efficient to train, are easier to interpret due to their mathematical basis, and work well in medical environments where textures are distinctly identifiable. 

This review compares various texture feature extraction methods and shows that wavelet features, when fed into a support vector machine (SVM) [30] learning model, achieved the highest performance in distinguishing between an abnormal and a normal breast (99% accuracy, 100% sensitivity, and 98% specificity) [31], as well as, in identifying type of lesion (accuracy of 99.17%, macro sensitivity 99.17%, and macro specificity: 93.45%) [32]. On the popular DMR-IR dataset, the best performance was achieved by extracting the Histogram of Oriented Gradient (HOG) [33] features fed into an SVM learning model achieving 98.27% accuracy, sensitivity of 97.19%, and specificity of 95.23%. This review also shows that while clinical trials are limited, they suggest that thermography’s performance is steadily increasing and comparable to mammography [34].

This review’s main contributions are as follows:

- A comprehensive review of recent advancements in texture analysis techniques and machine learning approaches specifically applied to breast cancer detection using infrared thermography, filling a gap in previous reviews that did not adequately emphasize texture analysis, and showing that this approach achieves top performance.
- A systematic analysis of the complete infrared thermography processing pipeline, including image preprocessing techniques, feature extraction methods, feature reduction techniques, classification approaches, and performance assessment metrics used in thermographic breast cancer detection, rather than focusing on isolated components of the workflow.
- A critical analysis of the current limitations in infrared thermography research, particularly noting the over-reliance on limited thermal image datasets (primarily from the DMR-IR database). This study also notes that some reported results may be unreliable due to potential leakage caused by splitting patient images between training and test sets which could contribute to model overfitting.
- The identification of promising research directions, highlighting how automated analysis through texture analysis and machine learning can address practical implementation challenges, such as the shortage of specialized radiologists and differing interpretation standards, bridging technical advances and clinical application. Numerous approaches achieved very high performance; therefore, this review advo-cates investing in research focused on developing tools that improve radiologists’ comprehension of medical images and the rationale behind CAD system recommendations.

This paper includes many equations describing the various textural approaches. The following Table 2 summarizes the key notations employed in this paper.

The rest of this paper is organized as follows: Section 2 presents a common processing pipeline for a computer-aided diagnosis (CAD) system to detect breast cancer in thermographic images; Section 3 presents methods and techniques to detect cancer using texture analysis; Section 4 discusses key gaps and worthy areas of future research, Section 5 discusses the key ideas presented in this review and Section 6 summarizes this review.

## 2. Computer-Aided Diagnosis System Architecture

Infrared thermography as a medical diagnostic tool has significantly improved in recent years due to the following factors: (1) thermal imaging is less sensitive to light, so poor illumination does not adversely affect the image; (2) temperature as a diagnostic tool has shown promising results in the early detection of various diseases, including breast cancer, rheumatoid arthritis, osteoarthritis, and Raynaud’s syndrome; (3) a thermal imager can easily obtain the surface temperature distribution of the object being studied over a wide area with just one click; (4) infrared thermography is a non-invasive, noncontact, and radiation-free imaging technique, ensuring that patients are not exposed to harmful radiation; (5) advances in thermal sensing equipment and software image processing technologies have made computer-aided diagnostic systems feasible and accurate [35].

**Figure 2:**
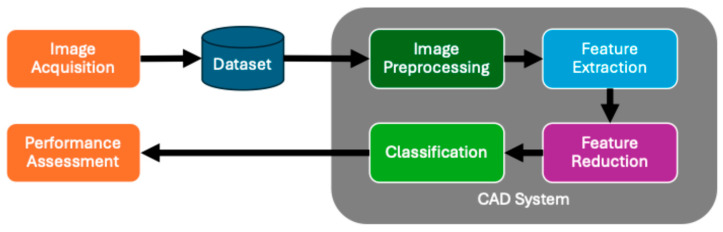
End-to-end process for a CAD system for breast cancer.

After decades of research into the use of infrared thermography in detecting breast cancer, many approaches have been proposed to automatically detect cancer lesions in these images. The overall approach applied in these cases is a workflow consisting of six steps as depicted in Figure 2.

Most CAD systems detect, and sometimes classify the type of abnormality, in one cycle through this workflow. Several proposed CAD systems operate in two cycles: (1) detect the presence of an abnormality; (2) classify the type of abnormality. De Freitas Barbosa et al. [32] created a two-stage classifier: (1) a classifier to detect the presence of a lesion (unhealthy); (2) a classifier to categorize the lesion identified by the first classifier into one of four types (healthy (no lesion), cyst, benign lesion, and malignant lesion), achieving 99% accuracy for the detection phase and 97.3% accuracy in the classification phase.

Image acquisition acquires the thermographic image in a controlled setting, often by a predefined protocol. The protocol includes rules for patient preparation, ambient room environment controls, views, and camera specifications [36,37,38]. Most research work in this area uses a previously acquired dataset, predominantly the publicly available DMR-IR dataset [39]. The predominant protocol employed captures static images of frontal, oblique, and lateral breast views. The reason for multiple perspectives is that a radiologist can better view deep lesions through a lateral or oblique view, rather than a frontal view. Dynamic Image Thermography (DIT) is an alternative protocol that cools the breast to a target temperature and takes multiple frontal images of the breast over time as it warms up [40].

Image preprocessing removes noise in the image introduced during the acquisition process, normalizes the image, improves image qualities important for accurate diagnostics, scales the image to a consistent size, and segments the image to only include the region of interest (ROI).

**Figure 3:**
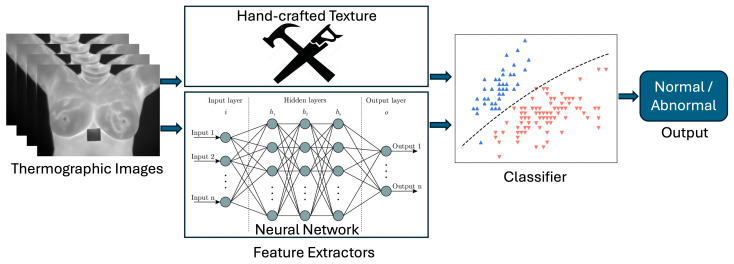
Combining hand-crafted textural with ANN-based feature extraction.

Feature extraction retrieves salient characteristics from the thermographic image using various texture extraction techniques, including statistical methods, model-based approaches, and signal analysis methods. Some approaches improve results by combining manually selected texture features with features extracted via a neural network. See Figure 3.

Feature reduction reduces the number of features by selecting discriminate features or mapping features to a lower-dimensional space.

The classification component is a machine learning model that is trained to determine whether a breast is normal or abnormal, and in some cases, the type of lesion. Most contemporary research is focused on supervised models that detect the presence of a cancerous lesion or abnormal breast. 

Performance assessment is the step that measures and reports the performance of the CAD system throughout its lifecycle: system development, clinical trials, and clinical use. The calculated metrics help radiologists and data scientists to assess the system’s effectiveness in identifying cancerous lesions and to identify scenarios where it underperforms. Various approaches and parameter fine-tuning can be analyzed to achieve optimal results.

Developing new CAD systems for the detection of breast cancer using thermographic images is an active area of research. The current methods are effective at identifying the presence of a cancerous lesion; however, key challenges with the current CAD systems are that they do not assign a risk score and lack sufficient clinical testing to enable real clinical use.

The next section will describe texture-based CAD systems.

## 3. Computer-Aided Diagnosis for Breast Cancer Detection

Most of the work on CAD systems designed to detect breast cancer has focused on identifying the presence or absence of cancer in the breast, and in some cases identifying the type of lesion. Upon identifying a cancerous lesion on a thermographic image, the radiologist often recommends a biopsy to confirm whether the tumor is benign or malignant, which requires isolating the precise location of the tumor. Thermography does not provide sufficient information for the radiologist to precisely locate the cancerous lesion for biopsy, and therefore, they must rely on other modalities such as ultrasound. Identifying cancerous lesion locations in thermographic images is a research area requiring further investigation.

### 3.1. Image Acquisition

Regardless of modality, in a clinical setting, images for breast cancer screening must be captured according to an approved standard. For mammography, MRI, and ultrasound, Breast Imaging Reporting and Data System (BI-RADS) is the standard for capturing images and reporting results for breast cancer screening. The American College of Radiology (ACR) created this standard in 1993 to standardize mammography findings and results [11] and subsequently extended it to support MRI and ultrasound. This standard is thorough in terms of assessment, classification system, terminology, reporting, and follow-up monitoring. It assesses the risk of developing breast cancer on a scale from 0 to 6 with 6 indicating a diagnosis confirmed by biopsy. 

In contrast, there is no common classification standard applied to thermographic images [12]. There are currently three standards: the Modified Ville Marie Infrared Scoring Scale [36] created by Dr. Keyserlingk in 1998, Guidelines for Breast Thermography published by the American Association of Thermology [37] (American Standard) which adopted and modified the Ville Marie standard, and the Standards and Protocols in Clinical Thermographic Imaging published by the International Academy of Clinical Thermology [38] (International Standard). Although the grading scales differ slightly, all the standards recommend that thermologists grade and compare vascular patterns in the two breasts, as well as with past thermographic images to identify changes over time. An increased vascular pattern and higher vascular asymmetry between breasts are associated with a higher risk of an abnormal condition. These grading scales are also not comparable to BI-RADS [37]. Table 3 compares the three standards. Although descriptions are similar between the three standards, their interpretation directions differ and lack the detail included in the BI-RADS standard.

The American Standard and International Standard include the following topics: pre-examination protocol, patient assessment, imaging system requirements, environmental controls, image capture protocol, image interpretation guide, reporting standard, follow-up protocol and thermologist education guidelines. The American Standard mandates that infrared imaging systems must capture images at a resolution of 640 × 480 pixels (307,200 pixels total), with a thermal sensitivity of 50 mK NETD (Noise Equivalent Temperature Difference) and a temperature precision of 0.05 °C. In contrast, the International Standard requires only 19,200 pixels, a thermal sensitivity of 80 mK NETD, and a temperature precision of 0.1 °C. It is important to note that all the imaging systems used to acquire the datasets listed in Table 4, except the FLIR A300, conform to the stricter American Standard.

Additionally, the American Standard also includes a guideline on the use of AI for image interpretation and identifies potential limitations to thermography, including extremely small or large breasts, menstrual cycle variations, hormone replacement therapy, breastfeeding, severe vascular diseases and difficulties detecting deep lesions in early-stage tumors.

#### Datasets

Numerous thermographic image datasets exist (See Table 4 and Tsietso et al. [28]), though the majority of them are not publicly available. Most of the images in these datasets are captured according to one of the three standards identified above.

When creating a new dataset, images are captured following a protocol, labeled, and stored in a digital format. Often, the labeling is limited to the identification of the patient’s breast as normal or abnormal and does not identify the location or type of abnormality. Datasets may include thermal matrices, grayscale images, and colorized images, typically in JPEG format, masks defining the region of interest (ROI) and clinical information. Important clinical information to collect includes age, date of assessment, body temperature at the start of assessment, and method of verifying diagnosis. A biopsy is the most reliable means of verification, although mammography is often employed as an alternative to biopsy due to cost and inconvenience. Therefore, using mammography may introduce a source of error. The report of the U.S. Preventive Services Task Force Screening for Breast Cancer Recommendation Statement [18,62] states that the mammography modality’s ability to detect breast cancer yielded a sensitivity for breast cancer that is approximately 77–95% and a specificity of 94–97%. For women with denser breasts, sensitivity drops to 63% and specificity to 90% in the worst case. To employ thermographic images in a research study, patients and an ethics committee must agree to their usage and define usage restrictions.

The two protocols for capturing thermographic images are Static Image Thermography (SIT) and Dynamic Image Thermography (DIT). SIT is the most prevalent standard, while DIT is evolving [40]. SIT captures bilateral frontal breast views, right and left mediolateral oblique breast views (30–45 degrees), and right and left lateral views of the breast in a temperature-controlled environment. In DIT, the patients’ breasts are cooled and frontal pictures are taken over time while the breasts warm up. The initial breast cooling better differentiates normal and abnormal breasts [40] because there is decreased vasoconstriction of blood vessels in abnormal breasts due to undeveloped muscles constricting the new blood vessels [63].

After image capture, the images are labeled and stored in a database. Most databases remain private lacking patient and ethics committee approval for wider distribution. The most prominent dataset used in research is the DMR-IR dataset which was publicly released in 2014 [39]. Another public dataset is the Breast Thermography at the Mendeley Data site [21,64] dataset which was released in 2024 and separately diagnoses right and left breasts, but the images are not compliant with the American Standard due to their lower resolution (320 × 240). Jalloul et al. [53] combined this dataset with the DMR-IR dataset to train the ResNet152 [65] Convolutional Neural Network (CNN) [66] feature extractor with an SVM classifier achieving 97.62% accuracy.

The unavailability of datasets is particularly challenging for the development of reliable models to classify breast cancer. Although there are private datasets that classify lesion types, there are no known public datasets that classify a thermographic image by lesion type. Table 4 lists the known datasets, showing that there are only four public datasets, and the DMR-IR dataset is the only public dataset with more than 100 patients.

**Figure 4:**
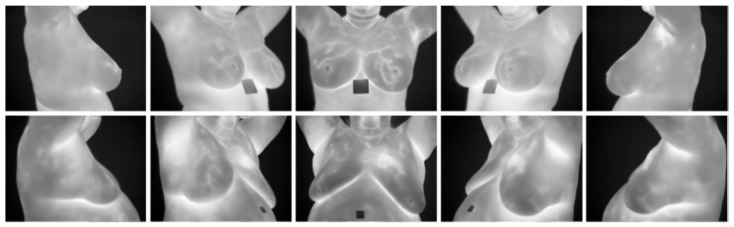
Images showing right lateral, right oblique, frontal, left oblique, and left lateral perspectives from the DMR-IR dataset [39]. The top row is images of patient #90, who is classified as healthy, and the bottom row is images from patient #180 who is classified as abnormal.

**DMR-IR Dataset:** The Database for Mastology Research Infrared (DMR-IR) dataset [39] is the most widely used database in research studies. Of the 26 studies covered in this review, 20, 77%, used this dataset. The DMR-IR dataset includes infrared (IR) images, several digitized mammograms, several ROI masks, and clinical data for 293 patients captured at the Hospital Universitario Antonio Pedro (HUAP) of the Federal University Fluminense. The use of this dataset was approved by the Ethical Committee of the HUAP and registered with the Brazilian Ministry of Health under number CAAE: 01042812.0.0000.5243 and is publicly available at http://visual.ic.uff.br/dmi/, accessed on 6 April 2025. Infrared images are captured using Static Image Thermography (SIT) and Dynamic Image Thermography (DIT) described in [19]. The database also includes segmented images for 56 patients (37 sick and 19 normal). Figure 4 shows sample images from this dataset.

Images and clinical data are recorded per patient exam, although there is only one exam for most patients. The exam summary includes a diagnosis of healthy or sick, determined by the assessment of mammographic images or by biopsy, but the specific modality for determining diagnosis is not specified.

Provided clinical data includes age, initial registration date, marital status, race, diagnosis (sick or healthy), complaints, signs, and temperature measured at the thorax at the time of image capture. Indicators are included for mammogram captured, radiotherapy performed, plastic surgery performed, prosthesis, hormone replacement, nipple changes (one or both breasts), and an indication of warts on the breast. Information was recorded in English or Portuguese and was not consistently coded. The images of sick breasts do not indicate if the abnormality is in the left or right breast, which is important information for performing asymmetric breast analysis.

Infrared images are captured with a FLIR SC 620 infrared camera [67] as 640 × 480 pixel images and provided in JPEG and thermal matrix format. Most jpeg images are provided in grayscale, but some are colorized, and some include a legend. Several digitized mammogram images are provided in grayscale format.

All diagnosis was validated by radiologists’ inspection of mammographic images, although 117 of the 293 patient’s diagnoses were also validated by biopsy (40%). Of the 105 patients classified as sick, 78 of them, 74%, were validated by biopsy. Preferring to perform biopsies on symptomatic patients is reasonable, given mammography’s high specificity of 94–97% [62] and the necessity to confirm a diagnosis, but is a factor to consider in any model trained on this dataset.

### 3.2. Image Preprocessing

Images captured by an infrared camera may contain grainy or speckled noise, often seen in dark areas, caused by variations in the thermal signals detected by the infrared sensor. Ambient room conditions and the status of the patient, such as sweating, hormonal differences, and menstrual cycle, impact image quality and consistency. The purpose of preprocessing is to remove noise, improve contrast, normalize intensity levels, and remove the background. Although infrared cameras capture infrared radiation measurements in a matrix of floating points representing their intensity level, cameras convert these matrices into a color image.

Most studies converted images to grayscale, but did not specify the mapping function. Dey et al. [41,42,43,54] created a grayscale image by extracting the red channel only because it contains higher temperatures, and therefore is indicative of an abnormality. In addition, most studies did not remove noise or improve image contrast. However, several studies applied an anisotropic diffusion filter [41,47,68] to remove noise while preserving edges and fine texture. In contrast, [45] applied a Gaussian filter, which tends to blur image lines. Two studies enhanced contrast by CLAHE [41,45]. Dey et al. [42] added noise (Gaussian, salt and pepper, and speckly nose) and showed that compressing the image before extracting first-order statistics improves model resiliency to noise, postulating that compressing removes high-frequency components.

**Figure 5:**
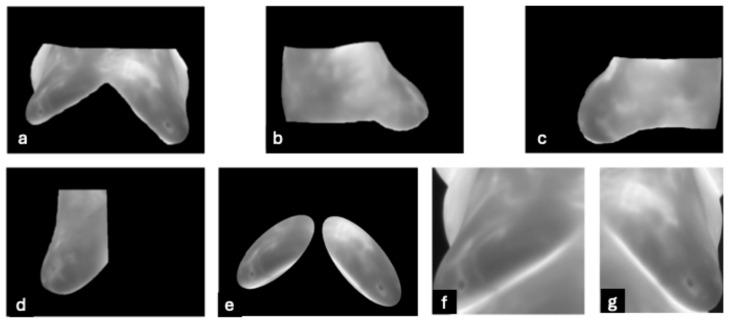
Segmentation approaches on patient #180 (abnormal) from DMR-IR dataset [39]: (**a**–**c**)—outline front, right, and left breast; (**d**)—pre-segmented right breast from DMR-IR dataset; (**e**)—outline breasts in same image; (**f**,**g**)—box right and left breasts.

Most thermographic images include the neck, shoulders, breast, and abdomen with a dark background (see Figure 1, Figure 4 and Figure 5). To ensure that background noise and non-breast areas do not distort results, many studies isolate the breast via segmentation. Several methods were employed, including segmenting a rectangular region encompassing each breast (Figure 5f,g), segmenting both breasts together (Figure 5a,e), and segmenting each breast separately (Figure 5b–d). Some studies separated the right and left breast in order to differentiate asymmetric temperature patterns between breasts indicative of a cancer lesion on one breast. Segmentation techniques include CNN, thresholding, region-based, edge-based, fully manual, and manually assisted.

Madhavi and Thomas [68] segmented the breasts using the Level-Set method [69] because it was simple to implement and robust in handling weak edges outlining both breasts in a single image; the results were similar to Figure 5a–c. Garia and Muthusamy [44] segmented 500 healthy and 500 sick patients captured by the DIT protocol from the DMR-IR dataset by proposing a dual-tree complex wavelet transform, a modified U-Net architecture. This model was trained on ground truth masks manually created by authors and achieved a Dice coefficient of 93.03%; the results were similar to Figure 5a–c. Code and masks are available at https://github.com/lalit2441/U-Net-data, accessed on 16 January 2025. Chebbah et al. [49] also applied the U-NET neural network to segment 90 images (60 normal and 30 abnormal) from the DMR-IR, similar to Figure 5e. Gonzalez-Leal et al. [45] created a large dataset containing 1793 patients by combining several smaller datasets, including the DMR-IR dataset. The images were segmented using a MXNet Resnet34 Neural Network [70] trained on ImageNet to segment each individual breast. Hakim and Awale [52], Dey [41,42,43,54], and Josephine et al. [61] segmented each breast with a rectangle, similar to Figure 5f,g. No study investigated the impact of segmentation on the results, although three of five studies without segmentation achieved high results [31,32,50]. While segmentation constrains the model to focus only on the region of interest and potentially enhance performance, its actual contribution remains uncertain without supporting ablation studies.

Although the Segment Anything Model (SAM) [71] has recently gained traction for the automatic segmentation of medical images, it was not applied in any of these studies. There is ongoing research to improve the segmentation of breast cancer lesions: Trongtirakul et al. [72,73] achieved leading performance with their segmentation approach on the DMR-IR dataset, with a pixel level accuracy of 97%, sensitivity 80%, and specificity 99%.

Several studies highlighted that a medically trained specialist must confirm breast segmentation, but it is difficult to determine whether segmentation is a key factor in performance. The best approach requires further study.

### 3.3. Feature Extraction

Texture features were extracted from thermographic images to identify abnormal breasts and, where possible, classify the type of cancerous lesion. A wide variety of texture features can be extracted, and the key challenge lies in identifying the most effective ones for classifying breast abnormalities. This section presents and summarizes the various texture features tested by recent studies.

This paper adopts the texture taxonomy defined by Tuceryan and Jain [74]: statistical, geometrical, model-based, and signal processing. Statistical methods capture information concerning the spatial distribution of gray values, geometrical methods capture texton spatial information, model-based methods create a model that describes or generates a texture, and signal processing methods represent texture by applying signal processing techniques. A section on geometrical methods was excluded because no study extracted geometrical texture features.

Each of the three textural feature sections includes a table with columns for texture feature names, references to studies that applied the method, advantages, and limitations. Many studies combined several texture features followed by a feature reduction step. Other studies focused on comparing texture features rather than combining them. In this case, the tables only show the best-performing feature selectors.

Several of the studies [41,42,43,44,54,75,76] assumed that cancer appears in only one of the two breasts, a condition known as unilateral breast cancer (UBC), and therefore, detect cancer by extracting and comparing texture features between the two breasts. An unhealthy breast’s texture pattern should be measurably different from a healthy breast’s texture pattern. Per Mejdahi et al. [77], the incidence of cancer appearing in both breasts, called synchronous bilateral breast cancer (SBBC), is rare and only occurs in 2.36% of the cases where cancer occurs in any breast. Therefore, asymmetric analysis of the breasts may be effective for detecting UBC, though it may fail to identify cases of SBBC. None of these studies have specifically tested their CAD system on SBBC cases, and therefore, asymmetric analysis on these cases is unknown. Furthermore, Dey et al. [41] noted that first-order statistics may not properly classify lactating breasts as temperature distributions may be asymmetric between breasts and cause false positives for techniques based on measuring differences between the left and right breast temperatures. However, this can be mitigated by identifying these cases during patient preparation.

It is noted that the Gray-Level Co-occurrence Matrix (GLCM) [78] is most frequently applied, appearing in 46% of the papers; however, Histogram of Oriented Gradients (HOG) [33] demonstrated superior performance.

#### 3.3.1. Statistical Methods

Statistical methods include first-order statistics (FOS), Co-occurrence Matrix techniques, non-parametric local transforms, and autocorrelation. No studies tested autocorrelation methods and therefore, this was excluded from this review. Table 5 summarizes the statistical methods covered in this review.

**First Order Statistics (FOS)**: This depends on individual pixel values and not on their interaction with other pixels. Captured statistics include entropy, energy, maximum, minimum, inter-quantile range, mean, standard deviation, mean absolute deviation, variance, range, root mean square, skewness, uniformity, and kurtosis [74,79]. Entropy measures the average level of information in an image, and therefore, breasts without cancer should have a lower entropy due to their homogenous temperature distribution, while a cancerous breast would have a higher entropy due to vascularization. Skewness measures distribution asymmetry, and therefore, a breast with cancer should show a higher skewness due to greater temperature values. See Table A1 in Appendix A for the first-order statistic equations.
**Tamura**: These are features that globally quantify six texture characteristics: coarseness, contrast, directionality, line-likeness, roughness, and regularity [80]. The equations for calculating these six features are in Table A2 in Appendix A.

Coarseness measures the scale of a textural pattern, where high coarseness represents a large pattern, and low coarseness represents a finer smaller pattern. This is measured by comparing the average intensity value captured at a radius of 2^k^ for all pixels in the image. Contrast measures the difference in intensity: high contrast has a high difference between low and high values while low contrast has a low difference between high and low values. This is measured by the ratio of the variance, a measure of dispersion, by the kurtosis, a measure of polarization. High contrast should have high variance and low polarization, while low contrast should have low variance and high polarization. Directionality identifies whether a pattern has a direction or no direction. Line-likeness identifies whether a texture is composed of lines. This is calculated by first creating the co-occurrence matrix $G_{Dd}\left(i,j\right)$ which is the normalized frequency of two different points separated by distance d where one point is associated with line coded i and the other point on line code j. Line-likeness is calculated per formula in Table A2 in Appendix A. Regularity measures whether a texture is regular or irregular. This is measured by assuming that any variousness in coarseness, contrast, directionality, and line-likeness is irregular. Roughness measures whether a surface is rough or smooth, which is measured by the sum of coarseness and contrast.

Mishra and Rath [79] included three of the Tamura features (coarseness, contrast, and directionality) with a number of co-occurrence matrices, but did not specify which Tamura features were retained during feature reduction, and therefore, its value cannot be determined.

**Figure 6:**
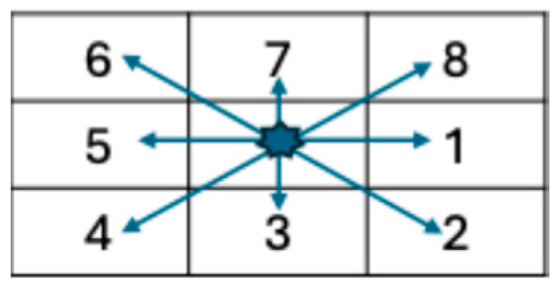
Neighborhood pixels of distance one from center pixel. Pixels 1 and 5 are at an angle $0^{{}^{\circ}}$, pixels 4 and 8 at an angle $45^{{}^{\circ}}$, pixels 7 and 8 at an angle $90^{{}^{\circ}}$, and pixels 6 and 2 at an angle $135^{{}^{\circ}}$.

**Co-occurrence Matrix**: This was developed by Haralick et al. [78] to codify textural information by calculating second-order statistics on the spatial relationships of gray tones in an image. This spatial relationship is captured in a matrix, called a Gray-level co-occurrence matrix (GLCM) of size LxL. Let g(a,b) represent an entry in the matrix that records the number of pixel pairs in image I that are separated by a specified angle and distance, where one pixel has a gray level of a and the other has a gray level of b. Figure 6 shows the neighboring pixels for all angles of distance 1.

Per Haralick et al. [78], fourteen second-order statistics are calculated on a normalized form of this matrix, where $p_{\theta d}\left(a,b\right)$ is the normalized version of g(a,b) such that θ is the specified angle and d is the specified distance between two pixels of intensity a and b, where 1≤a≤L and 1≤b≤L:

(1) $$p_{\theta ,d}\left(a,b\right)=\frac{g\left(a,b\right)}{\sum_{1\leq i,j\leq L}g\left(i,j\right)}$$

**Figure 7:**
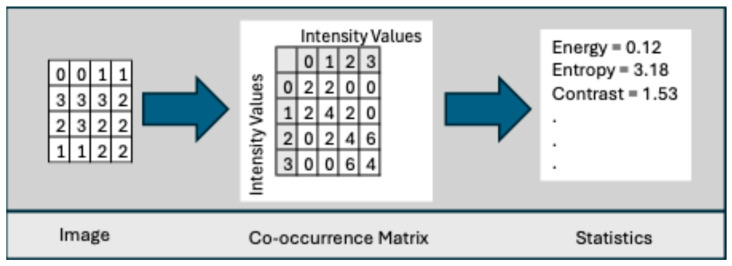
Example calculation of Haralick et al. [78] texture features with θ = 0 and distance = 1.

Furthermore, the following two equations calculate the probabilities that the summation and difference in the two-pixel intensities, represented by k, are as follows:

(2) $$p_{\theta ,d,x+y\;\;\;\;}\left(k\right)=\sum_{a+b=k}p_{\theta d}\left(a,b\right)$$

(3) $$p_{\theta ,d,x-y}\left(k\right)=\sum_{\left|a-b\right|=k}p_{\theta d}\left(a,b\right)$$

where x + y represents the summation of the two pixels and x − y represents the difference between the two pixels. Table A3 in Appendix A lists the texture features described in [78]. Figure 7 shows the process for a sample 4 × 4 image with four intensity levels.

Several studies computed additional order statistics on the GLCM, which are shown in Table A4 in Appendix A.

GLCM was tested and compared with other feature extraction methods in several studies. Resmini et al. [5] tested various combinations of GLCM with other feature extractors, including Local Ternary Pattern (LTP) [76], Daubechies Wavelet [90], and Higuchi and Petrosian Fractal Dimensions [91] and Hurst coefficients [92] and showed that GLCM performed best when measuring asymmetries between the left and right breast. A total of 80 patients (40 normal and 40 sick) captured using the SIT protocol were randomly selected from the DMR-IR dataset. The left and right breasts were manually segmented and converted to log-polar coordinates to register image pairs before the extraction of texture features. The features were fused and fed into a generic algorithm (GA) [93] for feature selection and classified with an SVM (RBF Kernel). The best-performing texture feature was GLCM alone, which achieved 94.61% accuracy, sensitivity 94.51%, specificity 94.87%, and AUC 94.87%.

Pereira et al. [57] selected 336 images from the HC-UFPE dataset and converted them from RGB to grayscale. Although the images included text and a color-bar scale, no preprocessing was performed to remove them. They applied SMOTE [94] to balance the class distribution, extracted GLCM second-order statistics and Zernike moments [95], and did not reduce the number of features using feature reduction techniques. They attempted to classify images as normal, benign lesion, malignant lesion, or cyst. The best result achieved an accuracy of 91.42% ± 2.93, macro sensitivity of 91.12%, and macro specificity of 91.36% with an SVM classifier. The sensitivity and specificity for normal, malignant lesion, and benign lesion exceeded 93%, but the sensitivity and specificity for benign lesions were 82.23% and 74.26%, indicating that the classifier struggled in differentiating benign lesions from other diagnoses. 

Rodrigues da Silva et al. [56] was published in the same year as Pereira et al. [57], adopted the same dataset and extracted the same features, but added feature selectors and tested more classifiers including Bayes net [96], naïve Bayes [96], multilayer perceptron (MLP) [96], SVM, decision tree [96], random tree [97], random forest [97], and Extreme Learning Machine (ELM) [98]. The best result was achieved with no feature reduction, and the ELM classifier achieved an accuracy of 94.00 ± 2.81. Instead of reporting sensitivity and specificity, they reported Kappa, a measure of agreement between predicted and observed categories, of 93.23% ± 0.03. In medical applications, sensitivity and specificity are the preferred metrics.

Josephine et al. [61] obtained images from screening camps that collected a total of 50 breast thermograms, 30 normal and 20 abnormal. RGB images were converted to grayscale and segmented under manual control by two observers. Four GLCM texture features and four statistical features were extracted and fed into several classifiers: Gaussian distribution analysis [99], K-nearest neighbor (KNN) [99], naïve Bayes, SVM, and AdaBoost models. AdaBoost achieved the highest accuracy of 91% and F1-score of 89%.

Chebbah et al. [49] extracted GLCM features and texture features representing blood vessels from each breast of 90 images selected from the DMR-IR dataset. Features from the left and right breast were fused and selected by applying a *t*-test and then fed into four classifiers: KNN, AdaBook, random forest, and SVM. The best results were obtained with an SVM classifier yielding an accuracy of 94.4%, sensitivity of 86.7% and specificity of 98.3%.

Gray-Level Run Length Matrix (GLRLM) [81,82] is a co-occurrence matrix that counts a sequence of pixels with the same intensity value in a specified direction. This technique captures statistical information about lines, which may represent blood vessels. Angiogenesis causes the growth of blood vessels, which are possibly detectable using GLRLM matrices.

Each value in the GLRLM co-occurrence matrix is indexed by the intensity value and the number of sequential pixels with that intensity value. Eleven different statistics capture information about short runs, long runs, nonuniformity, and other characteristic of the sequences; see Table A5 in Appendix A. Mishra et al. [79] calculated five additional second-order statistics on the GLRLM, which are shown in Table A6 in Appendix A.

Mishra and Rath [75] detected healthy and unhealthy breasts by asymmetric analysis of the right and left breasts of 56 patients (37 abnormal and 19 normal) from the DMR-IR dataset captured by the DIT protocol. Images were normalized and manually segmented, and GLCM and GLRLM features were extracted from each breast. Features whose difference between the average value of abnormal and normal exceeded a threshold of 0.15 were retained and fed into multiple classifiers. Features were retained from GLCM and GLRLM, indicating that both feature sets provided value: 8 of 22 GLCM features and 3 of 7 GLRLM features were selected. The best results were achieved with an SVM classifier, which achieved an accuracy of 97.03%, sensitivity of 94.22%, and specificity of 98.44% They extended their work [76] by selecting features with PCA and Autoencoder. A random forest classifier achieved the best in this study with an accuracy of 95.45%, sensitivity of 99.17%, and specificity of 88.07%. This method increased sensitivity at the cost of a much lower specificity, whereas the previous method yielded a more balanced sensitivity and specificity.

Three additional co-occurrence-based texture methods evaluated were the Gray-Level Size Zone Matrix (GLSZM) [85], Neighborhood Grey Tone Difference Matrix (NGTDM) [83], and Gray-Level Dependence Matrix (GLDM) [79,84]. Thibault et al. [85,100] developed the GLSZM technique as an alternative to GLRLM and reported that it performed better in identifying progeria disease in cell nuclei. It was modeled on the GLRLM co-occurrence matrix, except GLSZM counts the number of collocated pixels of the same intensity level in all directions. GLSZM’s co-occurrence matrix is indexed by intensity level and number of pixels in the area. Because it counts all the pixels of same intensity level in an area, a direction is not required. The same 11 statistics designed for GLRLM in Table A5 in Appendix A are employed for GLSZM, except the counts are based on areas, not run lengths. They introduced two additional statistics, Gray Level Variance and Zone Size Variance, to better differentiate large homogeneous zones that exhibit high intensity variation between them. Gray Level Variance is the square root of the Gray Level Variance equation in Table A6 in Appendix A and measures the variance between homogenous areas. Zone Size Variance is the square root of the Run Variance equation in Table A6 in Appendix A and measures the variance in zone sizes. Due to GLSZM’s focus on homogenous area size, it may be a good texture feature to distinguish cancer lesions as there should be smaller homogenous areas.

Neighborhood Grey Tone Different Matrix (NGTDM) [83] is considered a co-occurrence matrix method, although it represents information in a vector indexed by intensity level. The vector is calculated by adding the absolute difference in a pixel with all the neighboring pixels in the square of the side $\left(2d+1\right),\;d>0$ centered on the pixel to the vector indexed by the center pixel’s intensity value. Let $f\left(a,b\right)$ be a pixel of intensity value k and let d be the diameter of the square neighborhood of f(a,b), then $\bar{A_{k}(a,b)}$ is defined as the average of all the pixel values in the neighborhood of f(a,b):

(4) $$\bar{A_{k}(a,b)}=\frac{1}{{\left(2d+1\right)}^{2}-1}\sum_{i=-d}^{i=d}\sum_{j=-d}^{j=d}f(a+i,b+j),(i,j)\neq (0,0)$$

Let $N_{k}$ be defined as the set of all pixels of intensity k, except in the peripheral region of length d, then:

(5) $$N_{k}=\{f(i,j)|f\left(i,j\right)=k;\;d<i<M-d;\;d<j<N-d\}$$

Let $s\left(k\right)$ be the k^th^ entry in the vector, indexed by intensity value. Then $s\left(k\right)$ is defined as follows:

(6) $$s\left(k\right)=\sum_{f\left(a,b\right)\in N_{k}}\left|k-\bar{A_{k}\left(a,b\right)}\right|$$

$s\left(k\right)$ is calculated for all the pixels in the image, except in the peripheral region of width d. Five different statistics are derived from this vector: coarseness, contrast, busyness, complexity, and strength, which are defined in Table A7 in Appendix A. 

Gray-Level Dependence Matrix (GLDM) [79,84] is a co-occurrence matrix indexed by gray level and dependency count. It captures the number of times that the gray tone value difference between each pixel and a neighboring pixel is within a defined threshold, α. The set of neighboring pixels are all pixels that are of distance (Δx,Δy) from the central pixel, where Δx and Δy are defined values:

(7) $$neighbors=\{f(a\pm \Delta x,b\pm \Delta y)|f\left(a,b\right)\;is\;central\;pixel\;and\;\Delta x,\;\Delta y\;are\;defined\;values\}$$

All neighbors of $f\left(a,b\right)$, as defined in Equation (7), that satisfy the following equation are called dependent:

(8) $$\left|f\left(a,b\right)-f\left(\pm \Delta x,b\pm \Delta y\right)\right|\leq \alpha$$

The maximum number of neighbors for a specified gray level is defined as $N_{d}$. Table A8 in Appendix A lists the equations for the features extracted from a GLDM. Note that these equations are the same as shown for GLRLM in Table A5 and Table A6 in Appendix A, except the summation on the second index is based on the count of dependent pixels. 

Madhavi and Thomas [68] selected frontal, right, and left lateral images for 63 patients (31 abnormal and 32 normal) from the DMR-IR dataset and extracted GLCM, GLRLM, GLSZM, and NGTDM texture features. Features were selected using the *t*-test and then mapped to a lower dimensional feature space using kernel principal component analysis with a non-linear kernel function [99]. This reduced the size of the feature space from 150 to 16 features. Although features were selected from all four texture feature extractors, GLCM features showed the highest variance between normal and abnormal conditions. They trained a least square support vector machine (LSSVM) classifier with an RBF kernel on 36 patients and held out 27 for testing and achieved 96.3% accuracy, 100% sensitivity, and 92.3% specificity.

Mishra et al. [79] extended their work in [75,76] by adding features from the co-occurrence matrices GLSZM, NGTDM, and GLDM. A total of 92 features were extracted consisting of 14 features from first-order statistics, 24 features from GLCM, 16 features from GLRLM, 14 features from GLDM, 16 features from GLSZM, 5 features from NGTDM, and 3 features from Tamura. They achieved their best results with the SVM (RBF kernel) classifier with adaptive LASSO regression achieving accuracy of 96.79% and F1-score of 95.81%. Adding GLSZM, NGTDM, and GLDM features negatively impacted model performance, which was restored by adding the regressor. However, the paper did not indicate which features were retained by the regressor.

**Non-parametric local transforms**: rely on the relative ordering of local pixels, not on their intensity value. It includes Census Transform (CT) [86], Local Ternary Pattern (LTP) [76], Local Directional Number Pattern (LDN) [89], and local binary pattern (LBP) [87], which encode the local textural and structural properties of an image as binary codes.

**Figure 8:**
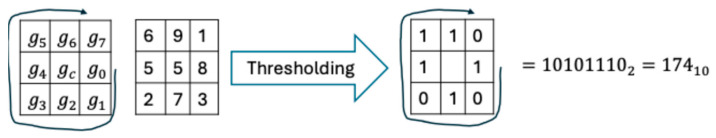
LBP operator on a 9 × 9 matrix. The center intensity value 5 is replaced by the intensity value 174.

LBP captures the textural information for a defined circle of radius R around each pixel. It derives a new image by replacing each pixel value in the original image with a new value that is derived by comparing it with a neighborhood of P (P > 1) pixels equally spaced from it on a circle of radius R > 0. Let $g_{c}$ be the intensity value of the center pixel and ${\{g}_{i}\left|\;i=0,1,\ldots ,P-1\right\}$ are the intensity values of the P pixels on the circle of radium R clockwise around $g_{c}$ (see Figure 8). Let

(9) $$s\left(g_{i}\right)=\left\{\begin{array}{c} 1,\;g_{c}\geq g_{i}\\ 0\;otherwise \end{array}\right.$$

Then the new pixel intensity value is calculated as follows:

(10) $$LBP_{PR}=\sum_{i=0}^{P-1}s\left(g_{i}\right)2^{i}$$

where $LBP_{PR}$ is the new intensity value for the center pixel. This operation is performed for all the pixels in the original image. See Figure 8.

In several cases, LBP performed well, but HOG outperformed it. Abdel-Nasser et al. [47] showed that LBP achieved 100% sensitivity, but a lower precision of only 82.7% and was outperformed by HOG. Garia and Muthusamy [44] showed that LBP outperformed GLCM features, but HOG performed best. Dihmani et al. [46] showed that LBP features performed better than the Canny edge detector and Gabor filter features, but HOG features achieved the best result. 

Similarly to LBP, CT determines new pixel intensity values based on Equations (9) and (10) described above, except for the set of points ${\{g}_{i}\}$ which is defined as all the points within a square of radius d centered on $g_{c}$. The advantage of CT over LBP is it captures more spatial and textural information. 

**Figure 9:**
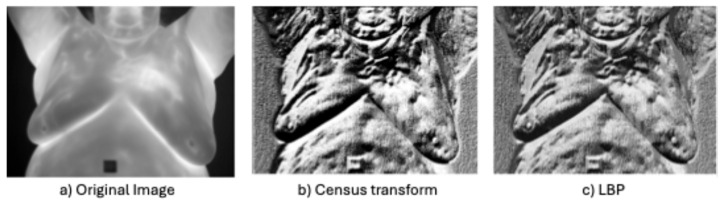
Comparison of a grayscale image of patient #180 (abnormal) from the DMR-IR dataset [39] with binary encoded CT and LBP setting R = 2 and P = 8.

Dey et al. [54] showed that CT outperforms LBP, theorizing that this was due to CT capturing more spatial information than LBP. Gonzalez-Lead et al. [45] combined LBP with FOS, GLCM, and HOG features and stated that the LBP features help to identify the hottest areas of the breast that might indicate the presence of a tumor due to increased temperature caused by vacuolization. See Figure 9 for an example of the original image and LBP and CT transformed images.

Resmini et al. [5] proposed an LTP transform replacing Equation (9) with the following:

(11) $$s\left(g_{i}\right)=\left\{\begin{array}{c} 0,g_{c}>g_{i}\\ 1,g_{c}=g_{i}\\ 2,g_{c}<g_{i} \end{array}\right.$$

Then the new intensity was calculated as follows:

(12) $$LTP_{PR}=\sum_{i=0}^{P-1}s\left(g_{i}\right)3^{i}$$

Eight central pixels intensities were calculated by rotating through eight different starting positions, $g_{0}$ through $g_{7}$ for radius R = 1. However, they showed that features extracted from a GLCM co-occurrence matrix significantly outperformed this version of LTP.

Pramanik et al. [51] proposed a non-parametric local transform which they tested on 226 patients from the DMR-IR dataset captured by the DIT protocol and tested on 120 images (50 healthy and 70 abnormal) from the DBT-TU-U dataset [59,60]. They selected the last image captured per patient in the DMR-IR dataset because, per da Silva et al. [39], the last image exhibits the best vascularization and hot regions [36]. To extract micro-level features, they developed a feature extractor, called the local instant-and-center-symmetric neighbor-based pattern of the extrema-images (LINPE), motivated by several non-parametric local transforms, including LBP, center-symmetric local binary pattern (CSLBP) [101], and local tri-directional pattern (LTriDP) [102] texture descriptors. They showed that their proposed approach outperformed texture features LBP [87], CSLBP [101], LTriDP [102], and Weber local descriptor (WLD) [103] feature extractors on the DMR-IR and DBT-TU-JI dataset.

Other non-parametric transforms have demonstrated better performance than LBP in various applications, but they have yet to be evaluated on thermographic images. Multi-scale Block Local Binary Pattern is an extended version of LBP developed by Liao et al. [104] that captures microstructural information similar to LBP but also captures macrostructural information. It outperforms LBP on face recognition. Zhao et al. [105] proposed Sobel-LBP which enhanced edge information before applying the LBP transform and showed that this technique outperformed LBP on face recognition.

#### 3.3.2. Model-Based Methods

Model-based includes random fields, fractal texture features and blood profusion models. There were no random field texture features extracted in the studies covered by this review and therefore were excluded. Table 6 summarizes the model-based methods covered in this review.

**Table 6:** Summary of model-based texture analysis methods employed in breast cancer detection.

Citation	Method	Features	Advantages	Limitations
[41,47,50,52]	Fractal [106]	Captures texture self-similarity patterns in the image. Includes fractal dimensions [91,107], Hurst exponent [92], and lacunarity [108].	Blood vessel growth is fractal [109]. Captures self-similar natural patterns. Invariant to rotation. Robust to illumination variations.	Inability to detect non-fractal patterns. Interpretability challenges. Sensitive to scale and noise.
[47,49,51]	Vascular Network	Models blood vessel development as a network.	Captures blood vessel network.	Accuracy of model. May require manual tuning.

**Fractal**: This represents texture as a self-similar pattern under varying degrees of magnification [110]. It was introduced into image processing by Pentland [106] and has been widely applied across image analysis, especially in medical image analysis [111]. Furthermore, many natural phenomena are fractal, including blood vessel growth and flow [109]. Therefore, fractal textures may prove effective in detecting cancer lesions in thermographic images.

Fractal dimension (FD) is a measure of the complexity, or space-filling, of a pattern. There are multiple methods to calculate FD, but the studies in this report used the Higuchi method [112], Petrosian method [113], or variations in the box-counting method to estimate the following equation [111]:

(13) $$FD=\log_{r\to 0}\left(\frac{\log\left(N_{r}\right)}{\log\left(\frac{1}{r}\right)}\right)$$

where $N_{r}$ is the number of nonoverlapping copies of a square of side r needed to cover an image subset. The Hurst exponent (HE) measures the self-similarity of an image. For self-affine process, it is linearly related to the fractal dimension by the following formula:

(14) $$HE=\left(n+1\right)-FD,\;where\;n=2$$

Lacunarity [108] measures the space or gap in a pattern. A low lacunarity is associated with homogeneous evenly spaced gaps while high lacunarity is associated with heterogenous gaps, i.e., more variation in size and distribution of gaps. To calculate lacunarity, define a square L of a specified size. Let p(m) represent the probability that there are m points within the square L centered about an arbitrary point and N is the maximum number of points in the square L. Lacunarity λ is defined as follows:

(15) $$\lambda =\frac{\prod_{m=1}^{N}m^{2}p\left(m\right)}{{\left(\sum_{m=1}^{N}mp\left(m\right)\right)}^{2}}$$

Dey et al. [41,42,43,54] tested multiple texture feature extraction and classification methods on two publicly available datasets to detect normal and abnormal breast pathologies. They created an 85-patient dataset by combining 69 patients from the DMR-IR dataset [39] and 16 patients from the Ann Arbor Thermography [55]. They utilized only the red channel, as cancerous regions tend to exhibit higher temperatures, which correspond to the red end of the color spectrum. The left and right breast were segmented into separate regions by applying Otsu’s thresholding [114] and seed growing. An anisotropic diffusion filter was used to remove noise while preserving edges and small structures [115] and contrast improved with CLAHE [116]. First-level statistics and fractal textures were extracted from each breast. Fractal features included fractal dimension [91,107] to identify self-similar patterns and Hurst exponent [92] to measure variance in pixel density. The spectral norm and Frobenius norm (Hilbert–Schmidt norm) were calculated for each breast and used to measure their similarity. Bilateral ratios were calculated for each feature and hard voting was performed across all the ratios to detect a patient with a breast abnormality. Fractal features outperformed statistical features, achieving an accuracy of 96.08% ± 3.87, sensitivity of 100 ± 0, and specificity of 93.57% ± 7.29 [41].

Moradi and Rezai [50] extract features from 200 healthy and unhealthy images from the DMR-IR dataset using the Segmentation Fractal Texture Analysis (SFTA) algorithm [117], which calculates fractal dimensions [91] from a set of binary images extracted from the image. After selecting features with the Firefly Algorithm [118] and Binary Grey Wolf Optimizer [119] in sequence, and classifying with a decision tree classifier, they achieved an accuracy of 97%, sensitivity of 98%, and specificity of 96%.

Abdel-Nasser et al. [47] propose a feature called Lacunarity Vascular Network that calculates the lacunarity on a vascular network extracted from the image by the method proposed in [120], but their ablation study showed that HOG outperformed it.

Hakim and Awale [52] extracted the Hurst coefficient [121], fractal dimension, and lacunarity features from 255 images selected from the DMR-IR dataset. Images were segmented where the ROI was a rectangle encompassing the breasts [12]. These features were fed into an SVM, logistic regression (LR), KNN, and naïve Bayes classifier to identify sick and healthy breasts. They achieved the highest accuracy with the naïve Bayes classifier achieving 94.53% accuracy and 97.75% specificity, but a lower sensitivity of 86.25%. 

Although the fractal extraction methods varied, Dey et al. [41] showed that fractal features outperformed FOS features and achieved 100% sensitivity while Moradi and Rezai [50] also achieved high results. Hakim and Awale [52] showed that lacunarity is insufficient to achieve high sensitivity. In summary, these studies show that the Hurst coefficients and fractal dimension features can effectively distinguish between normal and abnormal cases, whereas lacunarity was not effective.

**Vascular Network**: Several studies developed models to capture the vasodilation and angiogenesis of blood vessels. During preprocessing, Pramanik et al. [51] applied a breast blood profusion model based on breast thermal physiology which they developed and published in [26]. Chebbah et al. [49] applied thresholding and morphological operations (medial axis transformation) and a skeletonization algorithm called homotopic thinning to yield features representing blood vessels. Abdel-Nasser et al. [47] applied lacunarity analysis of Vascular Networks [120], but HOG outperformed it.

#### 3.3.3. Signal Processing Methods

Texture features can also be extracted using signal processing methods such as frequency transformation and filtering. This includes spatial domain filters, such as edge detection methods, Fourier domain filtering decomposing an image into its frequency components, and Gabor and wavelet analysis. None of the proposed methods included Fourier analysis of the image, but did employ edge detection, Gabor filters, and wavelets. Table 7 summarizes the signal processing methods covered in this review.

**Table 7:** Summary of signal processing texture analysis methods employed in breast cancer detection.

Citation	Method	Features	Advantages	Limitations
[46,122]	Spatial Domain Filters [74]	Obtain pixel value by applying operation to pixel neighbor. Used for edge detection and feature extraction. Includes Sobel [123], Canny [124] and HED [125].	Capture fine textures and edges.	Sensitive to noise. Does not capture course details.
[46]	Gabor Filter [126]	Captures spatial frequency texture information. Multi-scale and multi-orientation.	Capture course and fine detail. Spatial localization. Robust to illumination variations.	Sensitive to noise. Requires parameter tuning. Not rotational invariant. High dimensional vector.
[31,32,48,127]	Wavelet Analysis [90]	Represent textures spatially and frequency at multiple scales.	Capture course and fine detail. Spatial localization. Robust to illumination variations and noise.	Requires parameter tuning. Not rotational invariant. High dimensional vector.
[48]	Curvelet Transform [128]	Decomposes images into small, elongated wave-like shapes that capture details at different scales and orientations.	Identify vascular structures. Multi-scale and multi-orientation. Detection of curved edges.	Not in standard libraries. Not rotational invariant.
[29,44,45,46,47,127]	HOG [33]	Splits image into cells, calculates gradient/pixel and builds histograms of gradients/cells. Normalizes gradient in region of cells. Cells retain spatial detail.	Cells capture spatial detail. Identify shapes with distinct edges. Robust to illumination and geometric changes. Robust to noise and cluttered background. Detects abnormal structures in medical images.	Reliant on strong edge features. Dependent on manual choice of parameters. Output is a high-dimensional feature vector.

**Spatial Domain Filters**: Edge detection was employed by a few studies. Dihmani et al. [46] tested and compared the Canny edge detector [124] against HOG, LBP, and Gabor filters, but HOG achieved the best result. Youssef et al. [29] enhanced a thermographic with edges generated by the Canny and Holistically nested edge detector (HED) [125]. The HED is an end-to-end edge and boundary detector based on CNN.

Gama et al. [122] extracted, compared, and combined edge features extracted by the Canny edge detector [124] and HED applied to images in the DMR-IR dataset captured by the SIT protocol. The results of these edge detectors were fed into an Extreme Gradient Boosting [129] classifier. They achieved an accuracy of 97.4%, precision of 95%, recall of 100%, and AUC of 99%. The paper notes that HED outperforms Canny as a single feature, but Canny enhances the performance of HED.

**Figure 10:**
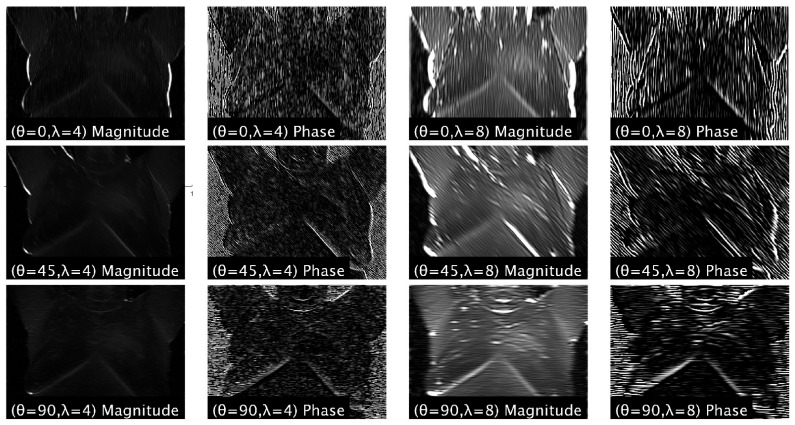
Applying a Gabor filter bank to a thermographic image of patient #180 (abnormal) from the DMR-IR dataset [39] with phases $0^{{}^{\circ}},\;\;45^{{}^{\circ}},\;and\;90^{{}^{\circ}}$ and pixel widths of 4 and 8.

**Gabor Filter**: This is a linear filter that identifies frequencies in a point’s localized area in a specified direction and is represented as a 2D Gaussian kernel modulated by a sinusoidal function [74]. The formula for the Gabor filter is as follows:
  (16) $$f\left(x,y\right)=e^{-\frac{1}{2}\left(\frac{x^{2}}{\sigma_{x}^{2}}+\frac{y^{2}}{\sigma_{y}^{2}}\right)}e^{-2\pi i\left(\mu_{0}x+\upsilon_{0}y\right)}$$

where $\sigma_{x}^{2}\;and\;\sigma_{y}^{2}$ are the variance of x and y, and $\mu_{0}\;and\;\upsilon_{0}$ are the centers of the sinusoidal function. See Figure 10 for an example of using the Gabor filter to extract features from an image.

Dihmani et al. [46] and Abdel-Nasser et al. [47] extracted Gabor filters and conducted ablation studies, but HOG outperformed them in both studies. Youssef et al. [29] enhanced a thermographic image by adding in texture features extracted using Gabor filters (see Figure 10).

**Wavelet Analysis**: Wavelets [90] are filters that decompose a signal in both space and time across a scale hierarchy. de Santana et al. [31] extract features using the Deep-Wavelet Neural Network (DWNN) based on the Haar Discrete Transform of Wavelets. They used 336 frontal images from the HC-UFPE dataset, which classifies an image as cyst, benign lesion, malignant lesion, or no lesion. The images were converted to grayscale and fed into DWNN and then classified with various classifiers. The best result was obtained with an SVM classifier with a linear kernel achieving an accuracy of 99.17%, macro sensitivity of 99.17%, and macro specificity of 93.45%. De Freitas Barbosa et al. [32] extended this work by adding a random forest feature selector, but they classified an image as normal (no lesion) or abnormal (cyst, benign lesion, or malignant lesion). They showed that DWNN outperformed InceptionV3 [130], MobileNet [131], ResNet-50 [65], VGG16 [132], VGG19 [132], and Xception [133] in the tasks of lesion detection and classification. The best result was obtained with an SVM classifier with a linear kernel achieving 99% accuracy, 100% sensitivity, and 98% specificity for lesion detection and 97.3% accuracy, 100% sensitivity, and 97% specificity for the lesion classification task.

Al-Rababah et al. [127] extract the Higher High (HH) band of the Discrete Wavelet Transform (DWT) from segmented images of 47 patients (31 abnormal and 16 normal) selected from the DMR-IR database acquired using the DIT protocol. The HH band is composed of high-frequency signals, which may capture information related to angiogenesis, potentially indicating a cancerous lesion. Oriented gradient distribution of the HH band for each image was captured by feeding the HH band into a HOG feature extractor. The number of features was reduced to 42 features/image by adding up 1000 consecutive HOG coefficients. To further reduce the number of features while retaining temporal changes in temperature, the feature mean was calculated for image pairs in the set of 20 DIT images for a patient. With an SVM classifier, they achieved an accuracy of 98.0%, sensitivity of 97.7%, and specificity of 98.7%.

**Figure 11:**
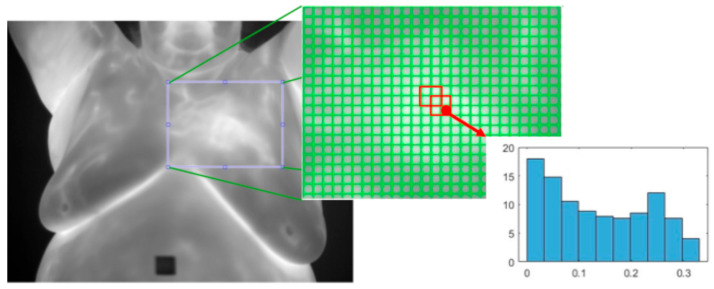
Frontal image of patient #180 (abnormal) from the DMR-IR dataset [39]. The explosion of the breast areas shows 8 × 8 cells as green boxes and the regions as red boxes of size 2 × 2 cells, which overlap adjacent regions by one cell. The normalized histogram of gradients for the area is provided as well.

**Curvelet transform**: This [128] decomposes an image into small, elongated wave-like shapes that capture details at different scales and orientations, particularly along curved edges. This technique may be particularly helpful in identifying the vascular structures associated with cancer. Karthiga and Narasimhan [48] applied a curvelet transform [128] to segment 60 thermographic frontal images (30 normal and 30 abnormal) from the DMR-IR dataset before extracting GLCM features. They also extracted first-order statistics, geometrical, and intensity features from the original images. Feature selection was performed using hypothesis testing and several machine learning models were subsequently compared. The best results were achieved with an accuracy of 93.3% and AUC of 94%. They also noted that the GLCM features extracted from the curvet domain increased accuracy by 10% points.
**Histogram of Oriented Gradients (HOG):** This [33] is a texture feature extractor that is also applied to the detection of objects in images. An image is split into non-overlapping cells of a predefined size. Regions are defined as a fixed number of cells and may overlap. The gradient is calculated for each pixel and the histogram of all the gradients within each cell is calculated. All the cell histograms of gradients within a region are normalized and concatenated into a single vector and then all the region vectors are concatenated into one vector. See Figure 11.

HOG offers several advantages: Its cell-based structure effectively captures spatial details, particularly shapes with distinct edges that may signify blood vessel growth. The normalization step improves robustness to illumination variations frequently observed in thermographic images. Additionally, HOG is resilient to geometric transformations such as rotation, scaling, and translation, enabling the detection of cancer lesions that can appear anywhere in the breast and vary in size and orientation.

A few of the studies conducted ablation studies of the extracted features and showed that HOG is the best-performing texture feature extractor. Dihmani et al. [46] extracted HOG, local binary pattern (LBP) [87], Gabor filters [126], and Canny edge detection [124] features from the pre-segmented images in the DMR-IR dataset (56 patients/1522 images). These features were then individually fed into multiple heuristic feature selection algorithms. The best result was achieved with the HOG features achieving an accuracy of 98.27% and F1-score of 98.15% while only using 25.78% of the HOG features. They further utilized the Shaplet Additive exPlanations (SHAP) [134,135] to quantify the importance of features which showed that the HOG feature distinctly separated abnormal and healthy thermographic images.

Garia and Muthusamy [44] confirmed that HOG features outperformed LBP, but also showed that it outperformed GLCM, although it performed slightly worse than features extracted using VGG-16. These features were extracted from 500 healthy and 500 sick patients captured by the DIT protocol from the DMR-IR dataset. After extracting features, the neighborhood component analysis (NCA) selected features for classification by the Kernel Extreme Learning Machine (KELM) [136] and random forest classifiers. The best result was achieved by extracting features with a VGG-16 neural network, selecting features with NCA, and classifying features with a random forest classifier, achieving an accuracy of 99.90%, although the HOG feature achieved an accuracy of 98.00%.

Gonzalez-Leal et al. [45] also showed that HOG outperformed GLCM and LBP in that order, although when combined they performed best.

Abdel-Nasser et al. [47] further confirmed that HOG outperformed LBP and GLCM, but also other feature extractors. They proposed a technique called learning-to-rank to model temperate deltas in a sequence of images captured by the DIT protocol. They obtained 56 pre-segmented patients from the DMR-IR dataset (37 abnormal and 19 normal) and extracted HOG, GLCM, lacunarity analysis of Vascular Networks [120], LBP [87], Local Directional Number Pattern [89], and Gabor filters from each image in the sequence and fed subsets of them into learning-to-rank to generate a single descriptive representation of the image sequence. The results were fed into an MLP classifier without applying feature reduction. The best result was achieved with the HOG texture feature achieving an accuracy of 95.8%, recall of 97.1%, precision of 94.6%, and F1-Score of 95.4%.

HOG feature extraction was also applied to the output of a Discrete Wavelet Transform (DWT). Al-Rababah et al. [127] used HOG to extract oriented gradient distribution from the Higher High (HH) band of the DWT from segmented images of 47 patients (31 abnormal and 16 normal) selected from the DMR-IR database acquired with the DIT protocol. With an SVM classifier, they achieved an accuracy of 98.0%, sensitivity of 97.7%, and specificity of 98.7%.

Dey et al. [43] extracted HOG features from the left and right breast, calculated their difference, and clustered the vectors into two groups using a K-mean cluster. The cluster with the higher medoid represented the abnormal cases and the lower medoid represented the normal cases. They achieved an accuracy of 86.25% ± 1.01, sensitivity of 87.22% ± 1.10, and F1-score of 85.83% ± 1.17, which is low compared to a supervised classifier.

Adding HOG features to a neural network feature extractor improved its performance. Youssef et al. [29] enhanced a thermographic image by adding in texture features extracted using Gabor filters, Canny edge detector, and HED edge detector. They extracted features from this enhanced image using a HOG feature extractor. In addition, they extracted features using two CNNs: ResNet-50 and MobileNet. ResNet is a residual network architecture designed to build deep networks while mitigating the vanishing gradient problem [65]. MobileNet is a lightweight network designed for efficient inference at low cost [131]. Together they bring together deep inferencing with shallow fast inferencing. Features were reduced with PCA and fed into an SVM and Extreme Gradient Boosting (EGB) [129] classifier. They conducted an ablation study, which individually achieved high results, but they achieved the best results by combining all three features (HOG, ResNet-50 and MobileNet). When combined, they achieved an accuracy of 96.22%, sensitivity of 97.19% and specificity of 95.23% using the EGB classifier.

### 3.4. Feature Reduction

After extracting features, many of the studies reduce the number of features by removing insignificant features, in a process called feature selection, or remapping the features to a lower dimensional feature space, in a process called dimension reduction. The purpose of this step is to improve the performance of the classifier component, reduce the overfitting of the model, speed up the training process, and identify the most discriminating features. This step could help radiologists interpret an image by identifying and prioritizing the most discriminate features. A feature may be correlated to another feature or consist mostly of noise, and therefore, should be eliminated to avoid bias in the classifier. Table 8 organizes the feature reduction methods employed by the studies in this review into four subcategories: feature selection, dimension reduction, and embedded and bio-inspired feature selection.

#### 3.4.1. Feature Selection

Feature selection’s purpose is to identify the most discriminative features that collaboratively maximize class separability between abnormal and normal classes, improving the performance of the classifier.

Two studies employed the *t*-test [137] to measure whether the mean of the features for the abnormal condition and normal condition is significantly different. Chebbah et al. [49] applied the *t*-test with a *p*-value of 0.01, eliminating four first-order statistics (energy, skewness, kurtosis, and smoothness) due to statistical insignificance, and retaining all the GLCM and blood vessel features. Madhavi and Thomas [68] applied the *t*-test with a *p*-value of 0.0001 to extract features from several co-occurrence matrices (GLCM, GLRLM, GLSZM, and NGTDM) reducing a total of 150 features to 45 features. These features were mapped to a reduced 16-dimensional feature space using Kernel PCA [99] with a polynomial diameter of 0.0005. Both studies selected local spatial features for discriminating between normal and abnormal breasts, appearing to capture the underlying physiology.

Random forest [138] is an ensemble learning model to select features based on their importance in distinguishing the target class, i.e., normal or abnormal. De Freitas Barbosa et al. [32] employed random forest to select features from DWNN and six CNNs. The best performance was achieved with the DWNN features, which the random forest feature selector reduced from 4096 features to 294 features. An SVM classifier with a linear kernel achieved very high results: 99% accuracy, 100% sensitivity, and 98% specificity for lesion detection. Although the model performed well, the paper does not quantify the value of the random forest feature selector.

Neighborhood component analysis [139] is a supervised feature subset selection model that weights feature importance by maximizing an objective function. Garia and Muthusamy [44] employed neighborhood component analysis to reduce 386 features extracted by LBP, GLCM, and HOG to 100 features. This eliminated irrelevant features without impacting results. They also applied neighborhood component analysis to a VGG-16 neural network, reducing 512 features to 100 features and marginally improving accuracy from 97.85% to 99.90%.

Pereira et al. [57] tested and compared multiple feature selection, dimension reduction, and bio-inspired methods. The feature selection methods included forward selection [97], correlation method [140], and objective dialectical method [141]. PCA was the only dimension reduction method tested and the bio-inspired methods included genetic algorithm [93], ant colony search [147], bee colony search [148], and particle swarm optimization [145] methods. They applied these feature reduction methods to a collection of RGB images from the HC-UFPE dataset, which were converted to grayscale. Images included text and color-bar scale, which may have distorted results. No preprocessing or segmentation was performed but they balanced the classes using SMOTE [94]. GLCM second-order statistics and Zernike moments [95] were extracted. The best result was achieved with no feature reduction, achieving an accuracy of 91.42% ± 2.93, macro sensitivity of 91.12%, and macro specificity of 91.36% with an SVM classifier. The Correlation Method did not reduce the number of features, while ODM achieved the highest accuracy among the feature reduction techniques (87.69% ± 3.21) with a 50% reduction in features; however, it still underperformed no feature reduction.

#### 3.4.2. Dimension Reduction

Dimension reduction maps a set of features into a new set of features in a lower dimensional space. Only three studies employed dimension reduction. Gonzalez-Leal et al. [45] reduced the dimension of the feature space by comparing kernel principal component analysis, independent component analysis [45], and locality-preserving projection [143]. They claimed that kernel principal component analysis achieved the best results, but did not include information on the dimension reduction or performance improvement gained by reduction in the feature space. Madhavi and Thomas [68] showed that kernel PCA reduced 45 features to 15 features. Pereira et al. [57] tested and showed that PCA did not improve results.

#### 3.4.3. Embedded

Embedded reduces the dimension of the feature space during training, typically through a regulation function.

Mishra and Rath conducted three studies between 2019 and 2024 [75,76,79] using the same images from the DMR-IR dataset. Their first study [80] extracted GLCM and GLRCM features with no feature reduction achieving an accuracy of 97.03% and F1-score of 95.50%. Their second study [81] added PCA dimension reduction, which reduced the number of features from 27 to 7 features. This reduced accuracy to 95.45% but increased F1-score to 96.66%. Their most recent study [79] incorporated first-order statistics, NGTDM, GLSZM, GLDM, and Tamura features; removed PCA; and tested two embedded feature selectors, including applied adaptive Least Absolute Shrinkage and Selection Operator (LASSO) regression [144]. They achieved their best results with the SVM (RBF kernel) classifier with adaptive LASSO regression achieving an accuracy of 96.79% and F1-score of 95.81%. The adaptive LASSO regressor improved accuracy by 17.36% and F1-score by 22.66%. Adding the new features negatively impacted model performance, which was restored by adding the regressor.

#### 3.4.4. Bio-Inspired

Bio-inspired methods simulate natural phenomena, such as gene inheritance and organism behavior, as well as validate generated gene subsets against an objective fitness function.

Moradi and Rezai [50] improved their results by selecting features with a Firefly Algorithm [118] followed by the Binary Grey Wolf Optimizer [119] in series. This improved accuracy from 77.5% with no feature reduction to 97% with feature reduction using a decision tree classifier. This also increased sensitivity from 76.1% to 98% and specificity from 78.9% to 96%. The feature selector also improved the accuracy of the SVM classifier by 84% and the KNN classifier by 88%.

Dihmani et al. [46] compared four metaheuristic feature reduction algorithms, including Binary Particle Swarm Optimization [150], Binary Spider Monkey Optimization [151], and two proposed hybrid algorithms. The paper proposed a hybrid algorithm that combined Binary Particle Swarm Optimization and particle swarm optimization [119] and a hybrid that combined Binary Spider Monkey Optimization and Spider Monkey Optimization [146]. The feature selectors were applied individually to Canny edge features, HOG features, Gabor filter features, and LBP features. The best result was achieved with the HOG features fed into the hybrid spider monkey optimization feature selector and classified with SVM, achieving an accuracy of 98.27% and F1-score of 98.15%, while only using 25.78% of the HOG features. However, the paper did not compare this result to a baseline without feature reduction.

Rodrigues da Silva et al. [56] utilized the same dataset (HC-EFPE), preprocessing, and feature extraction methods as Pereira et al. [57], but extended the approach by incorporating and comparing genetic algorithm and particle swarm optimization feature selectors, followed by classification using the ELM classifier with a polynomial kernel. Adopting this classifier improved the results by ~2.6% to an accuracy of 94.00% ± 2.8, but when adding the feature selector, performance degraded to 87.96% ± 3.77.

### 3.5. Classification

After the selection of the features, a model needs to be trained using the selected features to categorize the image. Ideally, the model should classify an image based on an accepted TH interpretation and classification system [37,38]; however, none of the datasets are labeled to this standard. The most popular dataset, DMR-IR, only classifies images as normal or sick (abnormal). Therefore, most of the models are binary classifiers and only classify an image as normal or abnormal.

Most of the studies tested multiple classifiers and compared the results. The tested classifiers include support vector machine (SVM), logistic regression (LR), kernel extreme learning machine, random forest, Bayes network, naïve Bayes, decision tree, multilayer perceptron, random tree, extreme learning machine, XGBoost, K-nearest neighbor, booster tree, bagged tree, AdaBoost, and Gaussian discriminant analysis. SVM was the most frequently evaluated classifier, appearing in 16 out of the 26 studies and achieving the best performance in 10 of them. Table 9 describes the classifiers that achieved the highest performance.

Overall, SVM generally outperformed other classifiers in comparative ablation studies. Three studies, Pereira et al. [57], Rodrigues da Silva et al. [56], and de Santana et al. [31], compared the performance of multiple classifiers on the HC-UFPE dataset, but differed in what features they extracted and selected for classification. All tested Bayes network, naïve Bayes, support vector machine, decision tree, multilayer perceptron, random tree, and random forest classifiers while two also tested the extreme learning machine. In two cases SVM performed the best, and in one case it performed slightly worse than the extreme learning machine. Karthiga and Narasimhan [48] also reported that the SVM classifier performed better than the decision tree, LR, KNN, boosted tree, and bagged tree on a subset of the DMR-IR dataset.

Pramanik et al. [51] proposed a classifier called LINPE-BL based on the Broad Learning (BL) network [154]: a two-layer neural network that uses tanh activation available at https://broadlearning.ai, accessed on 28 January 2025. They compared the results of their proposed approach with MLP and SVM classifiers and reported that their approach achieved an accuracy of 96.90% on the DMR-IR dataset and 94% on the DBT-TU-JI dataset, outperforming selected models and classifiers.

The only unsupervised approach was performed by Dey et al. [43] using k-mean clustering. Compared to supervised approaches, this underperformed. They clustered on the difference in HOG features between the left and right breast using various distance metrics within a k-mean cluster framework. The best result was achieved with the Spearman distance, which yielded 86.52% ± 1.01 accuracy, recall 87.22% ± 1.10, precision 89.21% ± 0.69 and F1-score 85.83% ± 1.17.

### 3.6. Performance Assessment

Evaluating system performance is important for several reasons: (1) to help tune the system and select parameters to optimize performance; (2) to confirm which features are of value in determining the presence of a cancerous lesion; (3) to determine if the system is ready for clinical use; (4) to confirm that production deployment operates within defined bounds.

Cross-validation is the predominant method employed in these studies to evaluate the performance of breast cancer detection systems. There are three types of cross-validation: (1) hold-out; (2) leave-one-out; (3) k-fold [97]. Hold-out reserves a set of test cases to independently test the system after training. This provides the best guarantee that the system will generalize well with new cases. However, it is important to note that many of these systems were trained and tested on a small dataset. A hold-out strategy on a small dataset would reduce the size of the training set, leaving a small test set leading to less reliable performance estimates and high variance in results. However, Gonzalez-Leal et al. [45] created a large dataset containing 1793 patients by combining several smaller datasets, including the DMR-IR dataset, but they only reported an AUC of 0.785. There could be various reasons for this low result, but without further information, we cannot determine the cause.

None of the studies applied the leave-one-out strategy and most studies applied a k-fold cross-validation strategy. This strategy randomly splits the dataset into k subsets: k-1 subsets for training, leaving one subset for testing. This is performed multiple times and results are reported as the mean of all the results and the confidence intervals. However, some studies did not report confidence intervals, and therefore, it is difficult to fully assess the model’s performance. Chebbah et al. [49] is an example.

For medical applications, the standard metrics to report are accuracy, sensitivity, specificity, and Area Under Curve (AUC). Sensitivity is the true positive rate, or recall, which is the ratio of correctly identifying an abnormal condition vs. all the known abnormal conditions. Specificity is the true negative rate, which is the ratio of correctly identifying a normal condition vs. all the known normal conditions. AUC quantifies the classifier’s ability to distinguish between true positive rate (sensitivity) and false positive rate (1-specificity) as a percentage. One-half of the studies reported sensitivity and specificity and most of the others reported precision, recall, and F1-score, which is reasonable, but not preferred for medical applications. See Table 10 for definitions of performance metrics. Note that true positive (TP) is the number of abnormal cases correctly classified as abnormal, true negative (TN) is the number of normal cases correctly classified as normal, false positive (FP) is the number of normal cases incorrectly classified as abnormal, and false negative (FN) is the number of abnormal cases incorrectly classified as normal.

A critical consideration when applying machine learning to medical applications is ensuring that all the data associated with a single patient is assigned to only one dataset—whether training, validation, or testing. Multiple images are captured for each patient during a thermographic evaluation. The DIT protocol captures multiple frontal images which are very similar, while the SIT protocol captures frontal and lateral views which provide different perspectives of the breast. Distributing images from the same patient across training, validation, and testing datasets would lead to information leakage. The classifier would learn features specific to that patient, which would improve the classification of the patient in the validation and testing datasets. This has the double effect of overfitting and improving performance on the test set. Nine studies [41,42,43,44,45,46,47,54,68] provided sufficient evidence that patient images were not distributed across different subsets. However, these studies still achieved high performance confirming that texture features are an effective approach for identifying breast cancer.

### 3.7. Key Studies Included in This Review

Table 11, Table 12 and Table 13 summarize the key studies investigated in this review subdivided by texture feature method and listed in chronological order. For studies that did not publish sensitivity and specificity, the reported metrics are shown. Most studies reported high accuracy, sensitivity, and specificity in the 90%+ range, and many of them trained and tested their systems on subsets of the publicly available DMR-IR dataset. The “Leaked” column indicates whether there is a potential risk of information leakage from the training set to the test set. A value of “No” means the study explicitly confirmed that all the patient data was properly assigned to either the training or test set. A “?” denotes that the paper did not specify whether patient-level separation was maintained. Due to the inconsistent selection of data and experimental setup, it is difficult to fairly compare reported performance metrics. To fairly compare performance results, data selection, and experiment setup must be consistent.

## 4. Future Directions

The studies reviewed in this survey evidence that thermography is a promising modality for detecting cancerous lesions in breasts. However, to achieve status as a primary modality, further research is required, and in the context of texture-based CAD systems, there are several areas worthy of further research work.

### 4.1. Benchmark

Over the years, extensive research has been conducted on breast cancer CAD systems that utilize thermographic images. Comparing the results to determine the best approach is difficult because studies did not adopt consistent datasets, protocols, or reporting standards. Datasets often did not classify images per standard and did not provide segmented images of the breast. To fairly compare different approaches, a standard evaluation framework is needed. This benchmark should include an extensive dataset of thermographic images organized by patient, captured and labeled according to a thermographic standard [37,38]; include segmented breast areas; identify the location of tumors; and include extensive clinical data. Ideally, the dataset should include multiple assessments for the same patient over an extended period, enabling the development of CAD systems that utilize temporal information to evaluate cancer risk. The dataset should include patients with varying temperature distributions caused by sweating, pregnancy, or hormonal imbalance. Where possible, the diagnosis should be confirmed by biopsy, but this may not be possible due to its cost and patient reluctance to undergo the procedure. Alternatively, diagnosis could be confirmed using a multi-model method that combines mammography, ultrasound, and MRI to improve diagnosis accuracy. Instead of attempting to label all images, alternative approaches include semi-supervised learning [155] and generative adversarial network (GAN) for synthesizing [156] or enhancing images [157]. Luo et al. [158] suggest a collaborative approach amongst multiple institutions called “federated learning” that would allow running models on patient data but protect their privacy.

### 4.2. Robust Clinical Trials

Although there are commercial CAD systems for detecting breast cancer currently available [159,160] that have participated in clinical trials [161], their use is still limited. Goni-Arana et al. [119] consolidated 22 clinical studies published between 2001 and 31 May 2023, evaluating the effectiveness of thermography for detecting breast cancer. They reported an average sensitivity of 88.5% and specificity of 71.8% across all the trials, noting that while these results are comparative to mammography, mammography potentially has a higher specificity and lower sensitivity. A key point they highlight is thermography specificity has been steadily improving over time. They believe that their study supports the continued use of mammography in high-income countries, while thermography is the most effective modality for developing countries. But, because the selected trials were composed of few patients, they recommend conducting more clinical studies to better determine thermography’s effectiveness. This confirms the FDA recommendation 43 years ago.

In concert with these clinical trials, radiologists need to agree on a single comprehensive interpretation standard in terms of assessment, classification system, terminology, reporting, and follow-up monitoring.

### 4.3. Improving Explainability for Radiologists

As mentioned in the introduction, the goal of a CAD System is to assist radiologists in making accurate diagnoses by effectively analyzing images while reducing the time required for evaluation. To accomplish this, the CAD system should provide information understandable by the radiologist. This includes recommending a TH or BI-RAD rating, identifying the suspected location of tumors, and identifying features that may help the radiologist to correctly interpret the image. Chebbah et al. [49] built a graphical user interface to help radiologists use and control their CAD. Dihmani et al. [46] applied Shaplet Additive exPlanations (SHAP) to quantify feature importance and then mapped the features back to a visual representation to help radiologists understand their recommendations.

Texture features may help a radiologist to interpret an image by enhancing the image. For example, Youssef et al. [29] extracted texture features to enhance thermographic images before classification. Texture features are typically based on well-defined mathematical models and are useful for building a CAD system but may not be directly understandable by a radiologist. To a radiologist, it is still a Blackbox. Translating these features into semantic concepts that allow a radiologist to reason about the thermographic image would help them to interpret the image differently and potentially lead to a more accurate diagnosis. Developing texture models that are more understood by the radiologist, such as the work around blood profusion models [47,49,50], needs further research.

### 4.4. Increasing Coverage

In some cultures, strict modesty norms may preclude women from exposing their breasts, even in a private medical setting. Moreover, access to breast screening technologies can be limited in remote areas. Enabling women to privately capture thermographic images of their breasts could offer a practical solution to these challenges.

Clinical trials are currently underway demonstrating the benefits of wearable digital health technologies, particularly in monitoring diabetes and cardiovascular diseases [162]. Yang et al. [163] provide an extensive overview of these technologies used in health and sports. Recent research has integrated temperature sensors into a wearable device for breast abnormality monitoring. Elouerghi et al. [164] developed the HealthCare Bra System (HCBS) as a portable non-invasive passive biomedical device that collects temperature data in real time from the skin surface of the breast. It is proposed as a remote device for breast cancer screening. This device is integrated into a bra, and therefore, in contact with the breast, enabling the simultaneous and real-time monitoring of skin temperature from any location on the breasts. Noise from the environment and camera are eliminated. Capturing temperature from multiple angles was explored by Francis et al. [165], who argued that multiple angles allow the detection of deeper cancer due to the effective depth limitation of thermography to 4.5 cm. Mammography and MRI are not appropriate techniques for a consumer device due to their use of X-rays and high-powered magnets. However, temperature sensor-based techniques are a promising area for the remote monitoring of breast cancer screening. They could provide a cost-effective replacement to SBE.

### 4.5. Multi-Modal Methods

The studies covered in this, and other reviews classify a breast abnormality by training models on images captured at a single session using a single image modality, such as mammography, thermography, infrared, MRI, or biopsy. However, each modality provides different insights about the patient and her breasts (See Table 1 in Introduction). Radiologists and oncologists use multiple modalities, including clinical information, biomarkers, and genomics captured over multiple visits to render an informed diagnosis. They recommend regular breast screening, with the interval between screenings varying based on factors including age, family history of breast cancer, biomarkers, and prior screening results. Therefore, a significant amount of information is collected about women’s health over many clinical visits, which is often saved in a health management system. However, there is limited research on how to structure this information and train a CAD system using it. 

However, there is research on integrating multiple modalities in a single CAD System. Pappachen and Dasarathy [166] survey the field of fusing multiple medical image modalities to improve image quality, increase clinical use of medical images, and improve diagnostic results. Integrating multiple modalities has been applied to various ultrasound modalities [167] and the classification of breast cancer by fusing pathological images with patient gene data [168]. Vanguri et al. [169] integrate radiological computed tomography images, tumor biopsies, and genomics to predict a patient’s response to immunotherapy for non-small cell lung cancer. Arena et al. [170] demonstrate how combining mammography, ultrasound, and thermography with clinical data could improve an earlier diagnosis of breast cancer. They describe two cases when thermography indicated cancerous regions earlier then mammography and explain how integrating thermographic information with mammographic images could have better highlighted the cancerous lesion, enabling earlier diagnosis by the radiologist. Although they captured mammographic and thermographic images for 2000 patients over 4 years, the results of these patients were not quantified in the report.

Radiologists will often use ultrasound as an adjunct modality to mammography, especially for younger women and women with denser breasts. Minavathi et al. [171] showed improved sensitivity when fusing features from mammographic and ultrasound images; however, they did not report specificity or accuracy.

Li et al. [172] developed LLaVA-Med, an adaptation of the GPT-4 foundation model tailored for a biomedical use case. A foundation model is a general class of models, typically based on the transformer neural network architecture, that is pretrained on broad and diverse datasets, generally using self-supervised learning at scale, and can be adapted to address the needs of specific applications [173]. Li et al. fine-tuned GPT-4 using image-text pairs of radiologic images and corresponding descriptions, enabling users to ask open-ended and closed-ended questions about the images. Although their model outperformed GPT-4 and other existing models, it still returned inaccurate responses, called hallucination.

### 4.6. Advances in Artificial Intelligence/Machine Learning

Classical neural networks have been employed to extract features [25,26,27], as well as texture features from thermographic images. For example, de Santana et al. [31] and De Freitas Barbosa et al. [32] implemented a wavelet called DWNN as a fixed parameter neural network and Gama et al. [122] implemented HED, a line extractor, as a neural network. There is limited work combining neural network features with texture feature extractors to improve performance. Youssef et al. [29] showed that combining a neural network with a texture feature extractor improves system performance over each individually. However, emerging artificial intelligence techniques are showing potential for application to breast cancer screening.

The Vision Transformer (VIT) deep learning model adapts the transformer model, originally designed for natural language processing, to image recognition tasks [174]. An image is split into flattened patches and fed into a transformer encoder, which includes a self-attention module, to capture relationships between image patches. Shamshad et al. [175] provide a comprehensive survey of ViT use in medical image analysis.

Capsule Network (CapsNet) is a deep learning model designed for image analysis that preserves the spatial hierarchy relationships often lost by CNNs. This capability makes them adept at recognizing complex patterns in an image, but are difficult to interpret and tend to be sensitive to small variations [176]. This model has been successfully applied to monkeypox [177].

A structured state-space model (SSM) is a deep learning model that has proved computationally efficient and able to model distant dependencies. It has been applied to image processing by marking image sequences with position embeddings and compressing the visual representation with bidirectional state space models [178]. Yue and Li [179] tested this model on 16 different medical datasets containing ten image modalities and 411,007 images; they achieved competitive results and provided access to their code at https://github.com/YubiaoYue/MedMamba, accessed on 21 May 2025.

Kolmogorov–Arnold Network (KAN) is an alternative deep learning model to the MLP that is composed of layers where learned weights are replaced by a univariate function parameterized by a spline and activation functions are learned [180]. Wang et al. [181] integrated KAN with ViT and tested it on 14 medical datasets containing various modalities. They showed that their model outperformed CNN, ViT, and MedMamba [179] image representation.

Quaternion Neural Networks (QNN) replace the real values processed by neural networks with quaternions, which are represented as four-dimensional vectors. QNNs have shown promising results across several use cases [182,183], including the classification of prostate cancer [184], pneumonia detection in chest X-ray images [185], and breast cancer detection in histopathological images [186]. Soulard and Carré [187] showed that representing a 2D wavelet transform as quaternions outperforms the real number version, which Greenblatt et al. [184] applied to the classification of prostate cancer by assigning a Gleason grade to a digital biopsy slide.

## 5. Discussion

Thermography was initially explored as a breast cancer screening modality in 1956 by Dr. Raymond Lawson, a Canadian surgeon [188]. In 1982, the FDA approved thermography as an adjunctive modality for breast cancer screening, but they have not approved it as a primary modality due to a lack of clinical evidence [4]. For many years, thermography was considered ineffective because infrared cameras were expensive and not sensitive enough for clinical use. But, over the years, the infrared camera sensitivity has dramatically improved at a more affordable price point [189].

Machine learning and AI pipelines have demonstrated the ability to deliver high-quality, cost-effective, and rapid analysis and classification. One class of pipelines that uses texture features as the key input data has been shown to be effective. Thermography images contain texture content that such AI pipelines can leverage.

This review establishes that texture-based CAD systems can accurately distinguish between abnormal and normal breasts, as well as identify lesion types, with high sensitivity and specificity. Many methods exceeded 90% accuracy, 93% sensitivity, and 90% specificity. High sensitivity is critical to reducing the risk of misclassifying a malignant lesion as normal (Type II error), while high specificity is important for preventing false positives (Type I errors), which can cause patient anxiety and lead to unnecessary diagnostic procedures.

This survey presented a CAD pipeline composed of six sequential stages: image acquisition, image preprocessing, feature extraction, feature reduction, classification, and performance assessment. In the first stage, image acquisition, the reviewed studies utilized various datasets following different image acquisition protocols. Although many of these datasets implemented predefined imaging procedures, none fully complied with the American or International Thermographic Standards. This lack of standardization introduces variability in imaging conditions, patient preparation, and environmental factors, complicating the comparison of results across studies.

Most studies tested their system on small datasets, the predominant choice being the publicly available DMR-IR dataset. To improve reproducibility and facilitate meaningful comparison, we recommend the development of a benchmark dataset based on a validated imaging standard that unifies the American and International Standards into a single guideline. While collecting such a dataset would require significant effort to implement and enforce the standard, a practical alternative could be to merge multiple existing datasets, as performed by Gonzalez-Leal et al. [45]. 

To address domain discrepancies and improve model generalization across multiple datasets, a segmentation technique can be applied to isolate the breasts by using one of the approaches reviewed in this survey, the Segment Anything Model (SAM) [71] or a segmentation method specifically designed to enhance cancerous regions, such as the method proposed by Trongtirakul et al. [72,73]. Furthermore, applying normalization techniques could reduce inter-dataset variability. These include spatial normalization (e.g., image resizing or anatomical landmark alignment) and thermal normalization (e.g., min-max scaling, z-score normalization, histogram equalization, CLAHE, or other normalization techniques). Notably, the most effective feature extraction methods - HOG and wavelet analysis- are robust to illumination, i.e., thermal, variation. This resilience suggests that these techniques may generalize well across heterogeneous datasets.

Furthermore, the private datasets typically did not specify how the diagnosis was validated. However, the most widely used dataset, DMR-IR, validated all the thermographic images via mammography and confirmed diagnosis via biopsy in 117 of the 293 (40%) patients. Of the 105 patients classified as having an abnormality, 78 of them, 74%, were confirmed by biopsy. Preferring to perform biopsies on symptomatic patients is reasonable, given mammography’s high specificity of 94–97% [62] and the necessity to confirm a diagnosis, but misclassifications may still exist and are a factor to consider in any model trained on these datasets.

The image preprocessing step typically converted images to grayscale and did not apply techniques to remove noise or improve contrast. Fifteen of the twenty studies segmented images to regions of interest that excluded areas outside the breasts. Although segmentation should improve model performance and robustness, its true impact remains unknown due to the absence of ablation studies. To accurately assess the impact of segmentation on model performance and robustness, ablation studies should be performed on larger datasets. The five remaining studies either deemed segmentation unnecessary or did not provide a rationale for omitting it.

A variety of texture analyses were tested in these studies, with Histogram of Oriented Gradients (HOG) and discrete wavelet-based features consistently outperforming other techniques, including some neural network-based feature extractors. Feature reduction was performed by most studies, applying a variety of techniques, including feature selection, dimension reduction, bio-inspired, and embedded. While several studies reported improved results, others showed no significant benefit. Overall, none of the reviewed feature reduction techniques consistently outperformed others. Although certain feature reduction techniques may be more effective, the current studies do not provide sufficient evidence to determine a clear preference.

Several classifiers were tested, with support vector machines (SVMs) demonstrating superior performance in comparative studies. In terms of performance, extracting wavelet features using a DWNN and fed into an SVM achieved the best performance distinguishing between an abnormal and a normal breast (99% accuracy, 100% sensitivity, and 98% specificity) [31] and identifying the type of lesion (accuracy of 99.17%, macro sensitivity of 99.17%, and macro specificity of 93.45%) [32]. In ablation studies, HOG outperformed the other texture methods, and on the DMR-IR database achieved the highest accuracy of 98.27% and F1-score of 98.15% [46]. However, HOG was not compared against DWNN, which achieved the highest result marginally better, with an accuracy of 99%. This study confirmed that all the images from each patient were assigned to a single data split (train, validate, and test), demonstrating that high performance is achievable without information leakage. Furthermore, this review shows that texture-based CAD systems are on par with neural network systems. De Freitas Barbosa et al. [32] showed that DWNN extracts wavelet texture features and outperforms several neural networks.

Explainability remains a critical challenge. Two studies [46,49] explored this aspect by incorporating features that facilitate radiologists’ understanding of the model’s recommendations. However, there is still a need for further research to improve and refine this capability.

Ethical considerations specific to the use of machine learning models for breast cancer classification are also important considerations. A major concern is data bias, which can arise when models are trained on datasets that lack sufficient size and diversity. To ensure fairness and accuracy, datasets should include a balanced representation of races, ages, physiological conditions, and other relevant factors. If a model produces an incorrect diagnosis, it raises the question of responsibility—should it lie with the radiologist, the developers, or the institution? There is also a risk that radiologists may become overly reliant on the model’s recommendations, potentially diminishing critical oversight. Finally, when models lack transparency in their diagnostic reasoning, patients may find it harder to participate meaningfully in their own care, potentially increasing anxiety and reducing trust in the diagnostic process.

Despite promising results, several limitations temper these findings. The selected studies lack ablation studies, inconsistent reporting of performance metrics, and insufficient diagnostic validation, particularly among studies using private datasets. Second, there is considerable heterogeneity among the reviewed studies in terms of imaging protocols, dataset characteristics, feature extraction techniques, classification models, and evaluation metrics. This variability makes direct comparison and synthesis of results challenging. Finally, few studies validate their models in clinical settings, and most do not account for physiological factors (e.g., menstrual cycle, hormonal therapy, and vascular conditions) that can affect thermographic patterns.

## 6. Conclusions

Computer-aided breast cancer diagnosis continues to be an active area of research. The CAD systems in this study have extracted texture features and show that they can achieve high results, and, in particular, on par with the results achieved by mammography as reported by the U.S. Preventive Service Task Force Screening for Breast Cancer Recommendation [62]. However, though promising, these results need more extensive clinical trial validation as the current trials and model testing have been limited to small datasets, which can lead to high variability and inaccurate results. This review suggests several worthy areas of research. Although there are a few thermographic-based systems commercially available, further research is required to effectively deploy texture-based CAD systems to monitoring centers and personal medical devices.

To advance the field, this survey recommends creating a large, standardized benchmark dataset based on unified imaging guidelines; conducting ablation studies; and improving clinical validation and reporting practices. Future work should also explore multimodal approaches that integrate thermography with other imaging modalities to enhance diagnostic accuracy.

## Figures and Tables

**Table 1:** Image modalities for breast cancer screening.

Modality	Description	Advantages	Limitations
Mammography	Uses low-dose X-rays on compressed breasts to identify abnormal growths such as tumors, cysts, or calcifications.	FDA approved as the primary modality. Appropriate for screening and diagnosis. Quick and non-invasive. Well-defined standard [11].	Less sensitive to women with dense breasts, especially in young women. Radiation exposure. Uncomfortable for women. Cannot use on pregnant women. Costly and less available in developing countries.
MRI	Uses a magnetic field and radio waves to create a detailed image of the breast after injecting the patient with IV contrast dye.	Detecting suspicious masses. Well-defined standard [11]. Greater sensitivity than mammography.	FDA approved as an adjunct modality. Expensive and less available in developing countries.
Ultrasound	Uses high-frequency sound waves to detect changes in the breasts. Used as an adjunct to mammography to detect and help classify abnormalities and guide biopsy.	East of use. Real-time imaging. Differentiates cysts from solid masses. Well-defined standard [11]. Sensitive to women with dense breasts. Can diagnose benign palpable masses. Can be used with breast implants. Radiation free. Painless and no discomfort. Can use on pregnant and lactating women.	FDA approved as an adjunct modality. Poor visibility to deep lesions.
Thermography	Uses thermal radiation emitted from breasts to detect differences.	Mobile and ease of use. Real-time imaging. Sensitive to women with dense breasts. Can be used with breast implants. Radiation free. Contactless and painless. Cost-effective for developing countries.	FDA approved as an adjunct modality. Multiple standards [12]. Patient temperature differences due to hormones, exercising, pregnancy, and menopausal cycle impact results. Requires temperature and humidity-controlled environment. Limited trials.

**Table 2:** Standard notation applied in this review.

Symbol	Description
I	Symbol representing an image.
L	Number of intensity levels in image I.
$f\left(x,y\right)$	Intensity level of pixel in image I at horizontal location 1≤x≤N and vertical location 1≤y≤M.
M, N	Number of rows and columns in image I.
$r_{k}$	The intensity levels for an L-level digital image, where k=0,1,2…,L−1.
$n_{k}$	Number of pixels in image f with intensity level $r_{k}$.
$h\left(r_{k}\right)$	$h\left(r_{k}\right)=n_{k}\;for\;k=0,1,\ldots ,L-1$ is the histogram of intensity values in f.
$p\left(r_{k}\right)$	$p\left(r_{k}\right)=\frac{h\left(r_{k}\right)}{MN}=\frac{n_{k}}{MN}\;for\;k=0,1,\ldots ,L-1$ is the normalized histogram of intensity values in f.

**Table 3:** Comparison of three grading standards. Although descriptions are similar, interpretation directions differ between the three standards.

Modified Ville Marie [36]		Thermobiological Grading System [37]		Twenty Point Thermobiological [38]	
Grade	Description	Grade	Description	Grade	Description
IR1	Absence of any vascular pattern to mild vascular symmetry	TH1	Symmetrical, bilateral, and nonvascular (non-suspicious, normal study)	TH1	Normal Symmetrical Non-Vascular
IR2	Significant buy symmetrical vascular pattern to moderate vascular asymmetry, particularly if stable	TH2	Symmetrical, bilateral, and vascular (non-suspicious, normal study)	TH2	Normal Symmetrical Vascular
IR3	One abnormal sign	TH3	Equivocal (low index of suspicious)	TH3	Questionable
IR4	Two abnormal signs	TH4	Abnormal (moderate index of suspicion)	TH4	Abnormal
IR5	Three abnormal signs	TH5	Highly abnormal (high index of suspicion)	TH5	Very Abnormal

**Table 4:** Summary of datasets.

Citations	Designer	Public	Classes	Protocol	Camera
[5,29,41,42,43,44,45,46,47,48,49,50,51,52,53]	DMR-IR [39]	Yes	Normal: 184, Sick: 105, Unknown:4	DIT, SIT	FLIR SC620
[41,42,43,54]	Ann-Arbor [55]	Yes	Normal: 4, Sick: 11, 15 images	SIT	Not specified
[31,32,56,57]	HC-UFPE [31,57]	No	Benign Lesion: 121, Malignant Lesion: 76, Cyst: 72, No Lesion: 66; 1052 images	SIT	FLIR S45
[53]	Mendeley [21]	Yes	Normal: 0, Benign: 84, Malignant: 35	SIT	FLIR A300
None known	Unnamed [58]	Yes	Normal: 6, High Risk: 2, Malignant: 11	SIT	Not specified
[51]	DBT-TU-JU [59,60]	No	Normal 45, Benign: 36, Malignant: 13, Unknown: 6	SIT	FLIR T650sc
[61]	Unnamed [61]	No	Normal: 30, Abnormal: 20	SIT	FLIR 74

**Table 5:** Summary of statistical texture analysis methods employed in breast cancer detection.

Citation	Method	Features	Advantages	Limitations
[45,48,49,61,79]	First-Order Statistics [74,79]	Estimate properties of individual pixels (mean, energy, entropy, kurtosis, etc.)	Statistical summary of intensity information. Computational efficient. Works well for homogenous images.	No spatial and local information. Ineffective for multi-texture images. Cannot identify lesion location.
[79]	Tamura [80]	Globally quantify coarseness, contract, directionality, likeness, roughness, and regularity.	Mimic human perception. Classifies texture. Scale invariant. Works well for homogenous images.	May not distinguish fine texture details. Ineffective for multi-texture images. Cannot identify lesion location.
[5,44,45,48,49,56,57,61,68,79]	Co-occurrence Matrix [78]	Capture frequencies of co-located values and calculate 2nd-order statistics (energy, entropy, contrast, homogeneity, etc.). Includes GLCM [78], GLRLM [81,82], NGTDM [83], GLDM [84], GLSZM [85], and GLDM [79,84].	Describes spatial relationships between pixels. Identifies surface pattern. Invariance to gray-level transformation.	Does not detect textures based on large primitives. Sensitive to scale and rotation. Restricted to a single direction. Dependent on manual choice of parameters
[44,45,46,51]	Non- parametric local transform [86]	Encodes local pixel intensity relationships. Includes LBP [87], CT [86], LTP [5,88], and LDN [89] texture features.	No probability distribution requirement. Robust to varying illumination. Captures local texture. Computational simplicity.	Noise sensitive. Limited global context. High dimensional vectors.

**Table 8:** Feature reduction methods.

Method	Cite	Type	Description
Feature Selection	[49,68]	*t*-test [137]	Select features with significant differences in class means.
	[32]	Random Forest [138]	Select features that maximally reduce impurity.
	[44]	Neighborhood Component Analysis [139]	Select features by maximizing an objective function.
	[57]	Forward Selection [97]	Add features until the target objective does not improve.
	[57]	Correlation Method [140]	Retain uncorrelated features.
	[57]	Objective Dialectical Method [141]	Select features that optimally balance relevance and redundancy.
Dimension Reduction	[45,57,68]	Principal Component Analysis (PCA) [99]	Reduce dimensions by mapping signals to orthogonal components and select those with the highest variance.
	[45]	Independent Component Analysis [142]	Map features to fewer statistically independent components.
	[45]	Locality Preserving Projections [143]	Preserves local structure in lower dimensional space.
Embedded	[79]	Adaptive LASSO Regression [144]	Select features by applying L1 regression penalizing absolute values of coefficients.
Bio- inspired	[57]	Genetic Algorithm [93]	Select feature subset evolved from feature population that maximizes fitness function.
	[46,57]	Particle Swarm Optimization [145]	Select features by simulating the collective movement of particles.
	[46]	Spider Monkey Optimization [146]	Select features by simulating the foraging behavior of monkeys.
	[57]	Ant Colony Search [147]	Select features by simulating the foraging behavior of ants.
	[57]	Bee Colony Search [148]	Select features by simulating the foraging behavior of bees.
	[50]	Binary Grey Wolf Optimizer [119]	Select features by simulating the behavior of grey wolves.
	[50]	Firefly Algorithm [149]	Select features by simulating the behavior of fireflies.

**Table 9:** Classifier methods that yielded the best results.

Classifier Method	Description
Support Vector Machine (SVM) [30]	Find a hyperplane that maximizes class separation.
Logistic Regression (LR) [96]	Maximum likelihood estimator of sigmoid function.
Decision Tree [96]	Recursive partition feature space to identify class.
Random Forest [97]	Train multiple trees on different feature and data subsets.
Multilayer Perceptron (MLP) [96]	A multilayered feedforward neural network with a non-linear activation function.
Naïve Bayes [96]	Identify class by maximum posterior probability.
AdaBoost [152]	Reduce misclassified instances by cascading multiple weak classifiers.
Least Square Support Vector Machine (LSSVM) [153]	Least square SVM that solves a set of linear equations instead of classical SVM technique.
Extreme Learning Machine (ELM) [98]	Single-layer feedforward neural network updating weights using Moore–Penrose pseudo-inverse.
Extreme Gradient Boosting (EGB) [129]	Reduce residual error by cascading weak decision trees.

**Table 10:** Performance measurement equations.

Description	Equation	Advantages	Limitations
Accuracy	$\frac{TP+TN}{TP+TN+FP+FN}$	Simple to understand and compute. Useful as a baseline metric.	Misleading for imbalanced datasets. Does not distinguish the type of error.
Sensitivity (Recall)	$\frac{TP}{TP+FN}$	Works for imbalanced datasets. Good metric for high-risk use cases. Compliments Precision and Specificity.	Ignores false positives, therefore may lead to high noise. Not complete alone.
Specificity	$\frac{TN}{TN+FP}$	Works for imbalanced datasets. Good metric for avoiding false alarms. Compliments Sensitivity.	Ignores false negatives, therefore may lead to high undetected positives. Not complete alone.
Precision	$\frac{TP}{TP+FP}$	Works for imbalanced datasets. Useful when positive is costly. Complements recall.	Ignores false negatives, therefore may lead to high undetected positives. May lead to under detection. Not complete alone.
F-Score	$\frac{2}{\frac{1}{Precision}+\frac{1}{Recall}}$	Works better for imbalanced datasets than accuracy. One metric that balances precision and recall. Widely adopted and understood.	Ignores true negatives. Hides precision and recall metrics.

**Table 11:** Selected systems for breast anomaly detection using statistical texture features.

Author(s)	Dataset	Leaked	Feature Extraction	Feature Reduction	Classifier	Performance
Madhavi and Thomas [68]	DMR-IR (63 patients)	No	GLCM, GLRLM, GLSZM, and NGTDM	*t*-test into Kernel PCA	LSSVM	Acc: 96% Sens: 100% Spec: 92%
Rodrigues da Silva et al. [56]	HC-UFPE (336 patients)	?	GLCM and Zernike moments	None	ELM	Acc: 94.00% ± 2.8 Kappa: 93.23% ± 3.1
Resmini et al. [5]	DMR-IR (80 patients)		GLCM	GA	SVM	Acc: 94.61% Sens: 94.61% Spec: 94.87%
Pereira et al. [57]	HC-UFPE (336 patients)	?	GLCM and Zernike moments	None	SVM	Acc: 91.42% ± 2.93 Macro Sens: 91.12% Macro Spec: 91.36%
Josephine et al. [61]	Private (50 images)	?	FOS and GLCM	None	AdaBoost	Acc: 91% F1-Score: 89%
Pramanik et al. [51]	DMR-IR (226 patients)	?	LINPE [51]	Training-based	LINPE-BL [51]	Acc: 96.9% Sens: 95.7% Spec: 97.2%
Chebbah et al. [49]	DMR-IR (90 images)	?	FOS, GLCM, and blood vessels	*t*-test	SVM	Acc: 92.2% Sens: 86.7% Spec: 98.3%
Mishra and Rath [79]	DMR-IR (56 patients)	?	FOS, GLCM, GLRCM, NGTDM, GLSZM, GLDM, and Tamura	Adaptive LASSO	SVM	Acc: 96.79% Precision: 98.77% Recall: 93.02% F1-Score: 95.81%

**Table 12:** Selected systems for breast anomaly detection using model-based texture features.

Author(s)	Dataset	Leaked	Feature Extraction	Feature Reduction	Classifier	Performance
Hakim and Awale [52]	DMR-IR (255 images)	?	HE, FD, and lacunarity	None	Naïve Bayes	Acc: 94.53% Sens: 86.25% Spec: 97.75%
Dey et al. [41]	DMR-IR (85 patients) Ann Arbor (16 patients)	No	HE and FD	None	Ensemble	Acc: 96.08% ± 3.87 Sens: 100% ± 0 Spec: 93.57% ± 7.29
Moradi and Rezai [50]	DMR-IR (200 images)	?	SFTA [117]	Firefly Algorithm to Binary Grey Wolf Optimizer	Decision Tree	Acc: 97% Sens: 98% Spec: 96%

**Table 13:** Selected systems for breast anomaly detection using signal processing textural features.

Author(s)	Dataset	Leaked	Feature Extraction	Feature Reduction	Classifier	Performance
Abdel-Nasser et al. [47]	DMR-IR (56 patients)	No	HOG	None	MLP	Acc: 95.8% Precision: 94.6% Recall: 97.1% F1-Score: 95.4%
Gonzalez-Leal et al. [45]	DMR-IR and others (1793 patients)	No	FOS, GLCM, LBP, and HOG	Kernel PCA	LR	AUC: 78.5%
Al-Rababah et al. [127]	DMR-IR (47 patients)	?	DWT into HOG	None	SVM	Acc: 98.0% Sens: 97.7% Spec: 98.7%
Karthiga and Narasimhan [48]	DMR-IR (60 patients)	?	FOS, GLCM, and curvelet transform to GLCM	Hypothesis testing	SVM	Acc: 93.3% AUC: 94%
de Santana et al. [31]	HC-UFPE (336 images)	?	DWNN	None	SVM	Acc: 99.17% Macro Sens: 99.17% Macro Spec: 93.45%
De Freitas Barbosa et al. [32]	HC-UFPE (336 images)	?	DWNN	Random Forest	SVM	Acc: 99% Sens: 100% Spec: 98%
Gama et al. [122]	DMR-IR (80 patients)	?	Canny edge and HED	None	EGB	Acc: 97.4% Precision: 95% Recall: 100% F1-Score: 97%
Garia and Muthusamy [44]	DMR-IR (1000 images)	No	HOG	NCA	Random Forest	Acc: 98.00% Precision: 97.05% Recall: 99:00% F1-Score: 98.01%
Dihmani et al. [46]	DMR-IR (56 patients)	No	HOG, LBP, Gabor filter, and Canny Edge	Hybrid Spider Monkey Optimization	SVM	Acc: 98.27% F1-Score: 98.15%
Youssef et al. [29]	DMR-IR (90 patients)	?	Image enhanced with Gabor filter, Canny edge, and HED to HOG fused with Resnet-50 + MobileNet	PCA	EGB	Acc: 96.22% Sens: 97.19% Spec: 95.23%

## Data Availability

No new data were created or analyzed in this study. Data sharing is not applicable to this article.

## Abbreviations

The following abbreviations are used in this manuscript:

Acc.	Accuracy
ACR	American College of Radiology
AUC	Area Under Curve
BI-RADS	Breast Imaging Reporting and Data System
CAD	Computer-aided diagnostic
CBE	Clinical breast exam
CSLBP	Center-Symmetric Local Binary Pattern
CT	Census Transform
DIT	Dynamic Image Thermography
DMR-IR	Database for Mastology Research Infrared
DWNN	Deep-Wavelet Neural Network
DWT	Discrete Wavelet Transform
ELM	Extreme Learning Machine
FD	Fractal Dimension
FDA	Food and Drug Administration
FOS	First-Order Statistics
GA	Genetic Algorithm
GAN	Generational Adversarial Networks
GLCM	Gray-Level Co-occurrence Matrix
GLDM	Gray-Level Dependence Matrix
GLRLM	Gray-Level Run Length Matrix
GLSZM	Gray-Level Size Zone Matrix
HE	Hurst Exponent
HED	Holistically nested Edge Detector
HH	Higher High
HOG	Histogram of Oriented Gradient
KAN	Kolmogorov–Arnold Network
KELM	Kernel Extreme Learning Machine
KNN	K-Nearest Neighbor
LASSO	Least absolute Shrinkage and Selection Operator
LBP	Local Binary Pattern
LDN	Local Directional Number Pattern
LINPE	Local instance-and-center-symmetric neighbor-based pattern
LINPE-BL	LINPE Broad Learner
LR	Logistic Regression
LSSVM	Least Square Support Vector Machine
LTP	Local Ternary Pattern
LTriDP	Local Tridirectional Pattern
MLP	Multilayer Perceptron
MRI	Magnetic Resonance Imaging
NCA	Neighborhood Component Analysis
NGTDM	Neighborhood Grey Tone Different Matrix
PCA	Principal Component Analysis
RBF	Radial Basis Function
ROI	Region of Interest
SBBC	Synchronous Bilateral Breast Cancer
SBE	Self-Breast Exam
Sens.	Sensitivity
SFTA	Segmentation Fractal Texture Analysis
SHAP	Shaplet Additive exPlanations
SIT	Static Image Thermography
Spec.	Specificity
SSM	Structured State-Space Model
SVM	Support Vector Machine
UBC	Unilateral Breast Cancer
ViT	Vision Transformer
WHO	World Health Organization
WLD	Web Local Descriptor

## Appendix A

The mathematical equations for the texture feature extraction techniques discussed in Section 3.3 are provided in this appendix.

**Table A1:** Equations for the first-order features.

Citation	Description	Equation	Citation	Description	Equation
[79]	Energy ^1^	$\sum_{0\leq k\leq L-1}p^{2}\left(r_{k}\right)$	[49,61,79]	Mean ($\mu_{I}$)	$\frac{1}{MN}\sum_{x,y}f\left(x,y\right)$
[45,47,48,79]	Min	$\underset{\left\{x,y\right\}}{\min}\left(f\left(x,y\right)\right)$	[49]	Smoothness [190]	$1-\frac{1}{1+\sigma_{I}^{2}}$
[48,79]	Max	$\underset{\left\{x,y\right\}}{\max}\left(f\left(x,y\right)\right)$	[79]	Interquartile Range	$\sum_{0.25L\leq k\leq 0.75L}p\left(r_{k}\right)$
[45,61,79]	Entropy	$\sum_{0\leq k\leq L-1}-p\left(r_{k}\right)\log\left(p\left(r_{k}\right)\right)$	[79]	Mean Absolute Deviation	$\frac{1}{MN}\sum_{x,y}\left|f\left(x,y\right)-\mu_{I}\right|$
[45,49,79]	Standard Deviation	$\sqrt{\sigma_{I}}$	[45,48,49,79]	Kurtosis $\left(\kappa \right)$	$\frac{\frac{1}{MN}\sum_{x,y}{\left(f\left(x,y\right)-\mu_{I}\right)}^{4}}{{\left(\sqrt{\sigma_{I}}\right)}^{4}}$
[79]	Range	$\underset{\left\{x,y\right\}}{\max}\left(f\left(x,y\right)\right)-\underset{\left\{x,y\right\}}{\min}\left(f\left(x,y\right)\right)$	[45,48,49,61,79]	Skewness	$\frac{\frac{1}{MN}\sum_{x,y}{\left(f\left(x,y\right)-\mu_{I}\right)}^{3}}{{\left(\sqrt{\sigma_{I}}\right)}^{3}}$
[79]	Root Mean Square	$\sqrt{\frac{1}{MN}\sum_{x,y}{f\left(x,y\right)}^{2}}$	[49,61,79]	Variance ($\sigma_{I})$	$\frac{1}{MN}\sum_{x,y}{\left(f\left(x,y\right)-\mu_{I}\right)}^{2}$

^1^ Mishra and Rath [79] call this equation uniformity. They define energy as $\sum_{x,y}{f\left(x,y\right)}^{2}$.

**Table A2:** Equations for the Tamura features.

Citation	Description	Equation
[79,80]	Coarseness ($F_{crs}$)	k = {1,2…h: h is a given positive integer} $A_{k}\left(x,y\right)=\sum_{i=x-2^{k-1}}^{x+2^{k-1}-1}\sum_{j=y-2^{k-1}}^{y+2^{k-1}-1}\frac{f\left(i,j\right)}{2^{2k}}\;\forall x,y,k\;E_{k,horiz}\left(x,y\right)=A_{k}\left(x+2^{k-1},y\right)-A_{k}\left(x-2^{k-1},y\right)\forall x,y,k\;E_{k,vert}\left(x,y\right)=A_{k}\left(x,y+2^{k-1}\;\right)-A_{k}\left(x,y-2^{k-1}\right)\forall x,y,k\;S_{best}\left(x,y\right)=2^{k}\;\;where\;k=\arg\underset{k}{\max}E_{k,d}\;\left(x,y\right)\forall k,d\in \left\{vert,\;horiz\right\}\;F_{crs}=\frac{1}{NM}\sum_{x}\sum_{y}S_{best}\left(x,y\right)$
[79,80]	Contrast ($F_{con}$)	$\frac{\sigma_{I}}{{\left(\kappa_{I}\right)}^{n}},\;where\;n\;is\;a\;positive\;number$
[79,80]	Directionality ^1^ ($F_{dir}$)	$\delta_{H}=convolve\;I\;with\left(\begin{array}{ccc} -1 & 0 & 1\\ -a & 0 & a\\ -1 & 0 & 1 \end{array}\right)$ $\delta_{V}=convolve\;I\;with\left(\begin{array}{ccc} 1 & a & 1\\ 0 & 0 & 0\\ -1 & -a & -1 \end{array}\right)$ $where\;a=1,2,\frac{1+\sqrt{5}}{2}\;\theta =\arctan\left(\frac{\delta_{V}}{\delta_{H}}\right)+\frac{\pi }{2};\;\left|\Delta G\right|=\frac{\left|\delta_{H}\right|+\left|\delta_{V}\right|}{2}\;N_{\theta }\left(k\right)=\left\{\#\;of\;points:\frac{\left(2k-1\right)\pi }{32}\leq \theta <\frac{\left(2k+1\right)\pi }{32},\Delta G>12\right\}\;H_{D}\left(k\right)=\frac{N_{\theta }\left(k\right)}{\sum_{i=0}^{15}N_{\theta }\left(i\right)},\;for\;k=0,1\ldots 15\;F_{dir}=1-rn_{p}\sum_{p=1}^{n_{p}}\sum_{\theta \in w_{p}}{\left(\theta -\theta_{p}\right)}^{2}H_{D}\left(\theta \right)\;\;\;r\;is\;normalizing\;factor;\;n_{p}number\;of\;peaks;\;{\;\;\theta }_{p}\;p^{th}\;peak\;position;\;w_{p}\;range\;of\;p^{th}\;peak\;between\;valleys$
[80]	Line-Likeness ($F_{lin}$)	$\frac{\sum_{i=1}^{n}\sum_{j=1}^{n}G_{Dd}\left(i,j\right)\cos\left|\frac{\left(i-j\right)2\pi }{n}\right|}{\sum_{i=1}^{n}\sum_{j=1}^{n}G_{Dd}\left(i,j\right)}$
[80]	Regularity ^2^	$1-r\left(\sigma_{F_{crs}}+\sigma_{F_{con}}+\sigma_{F_{dir}}+\sigma_{F_{ll}}\right)$
[80]	Roughness	$F_{crs}+F_{con}$

^1^ in [80], a = 1, but other values remain significant. ^2^ the function r(x) is called a normalizing factor in [80], but its meaning is undefined.

**Table A3:** Equations for the second-order statistics calculated using the GLCM based on Table 2 in Section 1 and Equations (1)–(3). These are the statistics from Haralick et al. [78]. References [56,57] extracted Haralick features but did not list which features were extracted and therefore are not cited in this table.

Citation	Description	Equation
[5,44,47,48,49,61,68,75,76,79]	Angular Second Momentum ^1^	$\sum_{a,b}p_{\theta ,d}^{2}\left(a,b\right)$
[5,44,47,48,49,68,75,76,79]	Contrast	$\sum_{a,b}{{\left(a-b\right)}^{2}p}_{\theta ,d}\left(a,b\right)$
[5,44,47,48,49,68,75,76,79]	Correlation	$\frac{\sum_{a,b}\left(\left(ab\right)p_{\theta ,d}\left(a,b\right)\right)-\mu_{x}\mu_{y}}{\sigma_{x}\sigma_{y}}$
[47,49,75,76,79]	Sum of Squares: Variance ^2^	$\sum_{a,b}{{\left(a-\mu_{x}\right)}^{2}p}_{\theta ,d}\left(a,b\right)$
[49,75,76,79]	Inverse Difference Moment	$\sum_{a,b}{\frac{1}{{1+\left(a-b\right)}^{2}}p}_{\theta ,d}\left(a,b\right)$
[47,68,75,76,79]	Sum Average ($f_{6}$)	$\sum_{k=2}^{2L}{kp}_{\theta ,d,x+y}\left(k\right)$
[47,68,75,76,79]	Sum Variance	$\sum_{k=2}^{2L}{{\left(k-f_{6}\right)}^{2}p}_{\theta ,d,x+y}\left(k\right)$
[47,68,75,76,79]	Sum Entropy	$-\sum_{k=2}^{2L}p_{\theta ,d,x+y}\left(k\right)\log\left(p_{\theta ,d,x+y}\left(k\right)\right)$
[47,49,68,75,76,79]	Entropy (H)	$-\sum_{a,b}p_{\theta ,d}\left(a,b\right)\log\left(p_{\theta ,d}\left(a,b\right)\right)$
[47,68,75,76,79]	Difference Variance	$\sum_{k=0}^{L-1}{{\left(k-f_{6}\right)}^{2}p}_{\theta ,d,x-y}\left(k\right)$
[47,68,75,76,79]	Difference Entropy	$-\sum_{k=0}^{L-1}p_{\theta ,d,x-y}\left(k\right)\log\left(p_{\theta ,d,x-y}\left(k\right)+\epsilon \right)$
[47,68,75,76,79]	Information Measures of Correlation	$\frac{H-HXY1}{\max\left\{HX,HY\right\}};\sqrt{1-e^{-2(HXY2-H)}},\;where\;p_{x}\left(i\right)=\sum_{j=1}^{L}p_{\theta ,d}\left(i,j\right);\;p_{y}\left(j\right)=\sum_{i=1}^{L}p_{\theta ,d}\left(i,j\right)\;HX=-\sum_{i=1}^{L}p_{x}\left(i\right)\log\left(p_{x}\left(i\right)\right);HY=-\sum_{j=1}^{L}p_{y}\left(j\right)\log\left(p_{y}\left(j\right)\right)\;HXY1=-\sum_{a,b}p_{\theta ,d}\left(a,b\right)\log\left(p_{x}\left(a\right)p_{y}\left(b\right)\right)\;HXY2=-\sum_{a,b}p_{x}\left(a\right)p_{y}\left(b\right)\log\left(p_{x}\left(a\right)p_{y}\left(b\right)\right)$
[79]	Maximal Correlation Coefficient	$\sqrt{second\;largest\;eigenvalue\left(Q\right)},\;where\;Q(i,j)=\sum_{k}\frac{p_{\sigma ,d}\left(i,k\right)p_{\sigma ,d}\left(j,k\right)}{p_{x}\left(i\right)p_{y}(k)}$

^1^ this is the same formula as energy in Table A1. ^2^ definition from [79].

**Table A4:** Equations for the second-order statistics calculated using the GLCM based on Table 2 and Equations (1)–(3).

Citation	Description	Equation
[47,68,75,76,79]	Autocorrelation	$\sum_{a,b}{abp}_{\theta ,d}\left(a,b\right)$
[61]	Contrast	$\sum_{a,b}{|a-b|p}_{\theta ,d}\left(a,b\right)$
[47,61]	Correlation	$\frac{\sum_{a,b}\left((a-\mu_{x})(b-\mu_{y})p_{\theta ,d}\left(a,b\right)\right)}{\sigma_{x}\sigma_{y}}$
[47,68,75,76,79]	Cluster Prominence	$\sum_{a,b}{{\left(a+b-\mu_{x}-\mu_{y}\right)}^{4}p}_{\theta ,d}\left(a,b\right)$
[47,68,75,76,79]	Cluster Shade	$\sum_{a,b}{{\left(a+b-\mu_{x}-\mu_{y}\right)}^{3}p}_{\theta ,d}\left(a,b\right)$
[79]	Cluster Tendency	$\sum_{a,b}{{\left(a+b-\mu_{x}-\mu_{y}\right)}^{2}p}_{\theta ,d}\left(a,b\right)$
[79]	Difference Average	$\sum_{k=0}^{L-1}{kp}_{\theta ,d,x-y}\left(k\right)$
[5,44,47,75,76]	Dissimilarity	$\sum_{a,b}|a-b|p_{\theta ,d}\left(a,b\right)$
[5,44,47,48,61,75,76]	Homogeneity I and II	$\sum_{a,b}\frac{p_{\theta ,d}\left(a,b\right)}{1+|a-b|};\sum_{a,b}\frac{p_{\theta ,d}\left(a,b\right)}{1+{\left(a-b\right)}^{2}}$
[79]	Inverse Difference	$\sum_{k=0}^{L-1}\frac{p_{\theta ,d,x-y}\left(k\right)}{k+1}$
[79]	Inverse Difference Normalized	$\sum_{k=0}^{L-1}1+\frac{k}{L^{2}}$
[79]	Inverse Variance	$\sum_{k=1}^{L-1}\frac{p_{\theta ,d,x-y}\left(k\right)}{k^{2}}$
[79]	Joint Average	$\sum_{a,b}ap_{\theta ,d}\left(a,b\right)$
[47,68,75,76,79]	Maximum Probability	$\underset{\left\{a,b\right\}}{\max}\left(p_{\theta ,d}\left(a,b\right)\right)$
[47,68,75,76]	Inverse Difference Normalized	$\sum_{a,b}\frac{p_{\theta ,d}\left(a,b\right)}{1+\frac{\left|a-b\right|}{L}}$
[47,68,79]	Inverse Difference Moment Normalized	$\sum_{a,b}\frac{p_{\theta ,d}\left(a,b\right)}{1+\frac{{\left(a-b\right)}^{2}}{L}}$

**Table A5:** Equations for the second-order statistics calculated using the GLRLM from [81,82]. Let $r_{\theta }(a,b)$ be defined as the number of runs in direction θ with pixel intensity value a and run length b such that $1\leq a\leq L\;and\;0\leq b\leq N_{r}$ where $N_{r}$ is the maximum run length possible. Let $n_{r}$ be the number of runs of different lengths and let $p_{\theta }\left(a,b\right)$ be the normalized version of $r_{\theta }(a,b)$, then $p_{\theta }\left(a,b\right)=\frac{r_{\theta }\left(a,b\right)}{\sum_{1\leq i\leq L}\sum_{0\leq j\leq N_{r}}r_{\theta }\left(i,j\right)}$.

Citation	Description	Equation	Citation	Description	Equation
[68,75,76,79]	Short Run Emphasis (SRE)	$\frac{1}{n_{r}}\sum_{i=1}^{L}\sum_{j=1}^{N_{r}}\frac{p_{\theta }\left(i,j\right)}{j^{2}}$	[68,75,76,79]	High Gray-Level Run Emphasis	$\frac{1}{n_{r}}\sum_{i=1}^{L}\sum_{j=1}^{N_{r}}p_{\theta }\left(i,j\right)i^{2}$
[68,75,76,79]	Long Run Emphasis (LRE)	$\frac{1}{n_{r}}\sum_{i=1}^{L}\sum_{j=1}^{N_{r}}p_{\theta }\left(i,j\right)j^{2}$	[68,79]	Short Run Low Gray-Level Emphasis	$\frac{1}{n_{r}}\sum_{i=1}^{L}\sum_{j=1}^{N_{r}}\frac{p_{\theta }\left(i,j\right)}{i^{2}j^{2}}$
[68,75,76,79]	Gray-Level Nonuniformity (GLN)	$\frac{1}{n_{r}}\sum_{i=1}^{L}{\left(\sum_{j=1}^{N_{r}}p_{\theta }\left(i,j\right)\right)}^{2}$	[68,79]	Short Run High Gray-Level Emphasis	$\frac{1}{n_{r}}\sum_{i=1}^{L}\sum_{j=1}^{N_{r}}\frac{p_{\theta }\left(i,j\right)i^{2}}{j^{2}}$
[68,75,76,79]	Run Length Nonuniformity (RLN)	$\frac{1}{n_{r}}\sum_{j=1}^{N_{r}}{\left(\sum_{i=1}^{L}p_{\theta }\left(i,j\right)\right)}^{2}$	[68,79]	Long Run Low Gray-Level Emphasis	$\frac{1}{n_{r}}\sum_{i=1}^{L}\sum_{j=1}^{N_{r}}\frac{p_{\theta }\left(i,j\right)j^{2}}{i^{2}}$
[68,75,76,79]	Run Percentage	$\frac{N_{r}}{NM}$	[68,79]	Long Run High Gray-Level Emphasis	$\frac{1}{n_{r}}\sum_{i=1}^{L}\sum_{j=1}^{N_{r}}p_{\theta }\left(i,j\right)i^{2}j^{2}$
[68,75,76,79]	Low Gray-Level Run Emphasis	$\frac{1}{n_{r}}\sum_{i=1}^{L}\sum_{j=1}^{N_{r}}\frac{p_{\theta }\left(i,j\right)}{i^{2}}$			

**Table A6:** Equations for the second-order statistics calculated using the GLRLM from [79].

Citation	Description	Equation	Citation	Description	Equation
[79]	Gray-Level Nonuniformity Normalized	$\frac{1}{n_{r}^{2}}\sum_{i=1}^{L}{\left(\sum_{j=1}^{N_{r}}p_{\theta }\left(i,j\right)\right)}^{2}$	[79]	Run Variance	$\sum_{i=1}^{L}\sum_{j=1}^{N_{r}}p_{\theta }\left(i,j\right){\left(j-\mu_{r}\right)}^{2}\;where\;u_{r}=\sum_{i=1}^{L}\sum_{j=1}^{N_{r}}{jp}_{\theta }\left(i,j\right)$
[79]	Run Length Nonuniformity Normalized	$\frac{1}{n_{r}^{2}}\sum_{j=1}^{N_{r}}{\left(\sum_{i=1}^{L}p_{\theta }\left(i,j\right)\right)}^{2}$	[79]	Run Entropy	$-\sum_{i=1}^{L}\sum_{j=1}^{N_{r}}p_{\theta }\left(i,j\right)\log(p_{\theta }\left(i,j\right))$
[79]	Gray Level Variance	$\sum_{i=1}^{L}\sum_{j=1}^{N_{r}}p_{\theta }\left(i,j\right){\left(i-\mu_{g}\right)}^{2}\;\;where\;u_{g}=\sum_{i=1}^{L}\sum_{j=1}^{N_{r}}{ip}_{\theta }\left(i,j\right)$			

**Table A7:** Equations for the second-order statistics calculated using the NGTDM. G is defined as the number of distinct gray levels in the image and $p\left(i\right)=\frac{N_{i}}{\left(N-2d)(M-2d\right)}$ is the probability of intensity k in the image areas that exclude a border region of width d.

Citation	Description	Equation
[68,79]	Coarseness	$\frac{1}{\epsilon +\sum_{i=0}^{L-1}p(i)s(i)},\;\epsilon \;prevents\;divide\;by\;0$
[68,79]	Contrast	$\left(\frac{1}{G(G-1)}\sum_{i=0}^{L-1}\sum_{i=0}^{L-1}p\left(i\right)p\left(j\right){\left(i-j\right)}^{2}\right)\left(\frac{1}{\left(N-2d)(M-2d\right)}\sum_{i=0}^{L-1}s(i)\right)$
[68,79]	Busyness	$\frac{\sum_{i=0}^{L-1}p\left(i\right)s(i)}{\sum_{i=0}^{L-1}\sum_{j=0}^{L-1}ip\left(i\right)-jp(j)},\;p\left(i\right)\neq 0,\;p\left(j\right)\neq 0$
[68,79]	Complexity	$\frac{1}{\left(N-2d)(M-2d\right)}\sum_{i=0}^{L-1}\sum_{i=0}^{L-1}|i-j|\frac{p\left(i\right)s\left(i\right)+p\left(j\right)s\left(j\right)}{p\left(i\right)+p\left(j\right)}$
[68,79]	Strength	$\frac{\sum_{i=0}^{L-1}\sum_{i=0}^{L-1}\left(p\left(i\right)+p\left(j\right)\right){\left(i-j\right)}^{2}}{\epsilon +\sum_{i=0}^{L-1}s(i)},\;\epsilon \;prevents\;divide\;by\;0$

**Table A8:** Equations for the second-order statistics calculated using the GLDM from [79]. Let g(a,b) be the co-occurrence matrix where the index a is the gray level and the index b is the number of dependent pixels on that gray level. $p\left(a,b\right)=\frac{g\left(a,b\right)}{\sum_{1\leq i\leq L}\sum_{1\leq j\leq N_{d}}g\left(i,j\right)}$ is the normalized version of g(a,b), where $1\leq a\leq L\;and\;1\leq b\leq N_{d}$.

Citation	Description	Equation	Citation	Description	Equation
[79]	Small Dependence Emphasis	$\frac{1}{N_{d}}\sum_{i=1}^{L}\sum_{j=1}^{N_{d}}\frac{p\left(i,j\right)}{i^{2}}$	[79]	Dependence Entropy	$-\sum_{i=1}^{L}\sum_{j=1}^{N_{d}}p\left(i,j\right)log(p\left(i,j\right)+\epsilon )$
[79]	Large Dependence Emphasis	$\frac{1}{N_{d}}\sum_{i=1}^{L}\sum_{j=1}^{N_{d}}p\left(i,j\right)j^{2}$	[79]	Low Gray Level Emphasis	$\frac{1}{N_{d}}\sum_{i=1}^{L}\sum_{j=1}^{N_{d}}\frac{p\left(i,j\right)}{i^{2}}$
[79]	Gray-Level Nonuniformity	$\frac{1}{N_{d}}\sum_{i=1}^{L}{\left(\sum_{j=1}^{N_{d}}p\left(i,j\right)\right)}^{2}$	[79]	High Gray Level Emphasis	$\frac{1}{N_{d}}\sum_{i=1}^{L}\sum_{j=1}^{N_{d}}p\left(i,j\right)i^{2}$
[79]	Dependence Nonuniformity	$\frac{1}{N_{d}}\sum_{j=1}^{N_{d}}{\left(\sum_{i=1}^{L}p\left(i,j\right)\right)}^{2}$	[79]	Small Dependence Low Gray Level Emphasis	$\frac{1}{N_{d}}\sum_{i=1}^{L}\sum_{j=1}^{N_{d}}\frac{p\left(i,j\right)}{i^{2}j^{2}}$
[79]	Dependence Nonuniformity Normalized	$\frac{1}{N_{d}^{2}}\sum_{j=1}^{N_{d}}{\left(\sum_{i=1}^{L}p\left(i,j\right)\right)}^{2}$	[79]	Small Dependence High Gray Level Emphasis	$\frac{1}{N_{d}}\sum_{i=1}^{L}\sum_{j=1}^{N_{d}}\frac{p\left(i,j\right)i^{2}}{j^{2}}$
[79]	Gray-Level Variance	$\sum_{i=1}^{L}\sum_{j=1}^{N_{d}}p\left(i,j\right){\left(i-\mu_{c}\right)}^{2}\;where\;u_{c}=\sum_{i=1}^{L}\sum_{j=1}^{N_{r}}ip\left(i,j\right)$	[79]	Large Dependence Low Gray Level Emphasis	$\frac{1}{N_{d}}\sum_{i=1}^{L}\sum_{j=1}^{N_{d}}\frac{p\left(i,j\right)j^{2}}{i^{2}}$
[79]	Dependence Variance	$\sum_{i=1}^{L}\sum_{j=1}^{N_{d}}p\left(i,j\right){\left(j-\mu_{d}\right)}^{2}\;where\;u_{d}=\sum_{i=1}^{L}\sum_{j=1}^{N_{r}}jp\left(i,j\right)$	[79]	Large Dependence High Gray Level Emphasis	$\frac{1}{N_{d}}\sum_{i=1}^{L}\sum_{j=1}^{N_{d}}p\left(i,j\right)i^{2}j^{2}$
